# Real‐time detection of N‐end rule‐mediated ubiquitination via fluorescently labeled substrate probes†

**DOI:** 10.1111/nph.14497

**Published:** 2017-03-09

**Authors:** Augustin C. Mot, Erik Prell, Maria Klecker, Christin Naumann, Frederik Faden, Bernhard Westermann, Nico Dissmeyer

**Affiliations:** ^1^ Independent Junior Research Group on Protein Recognition and Degradation Leibniz Institute of Plant Biochemistry (IPB) Weinberg 3 Halle (Saale) D‐06120 Germany; ^2^ ScienceCampus Halle – Plant‐based Bioeconomy Betty‐Heimann‐Str. 3 Halle (Saale) D‐06120 Germany; ^3^ Department of Bioorganic Chemistry Leibniz Institute of Plant Biochemistry (IPB) Weinberg 3 Halle (Saale) D‐06120 Germany

**Keywords:** activity profiling, E3 ligases, fluorescent dyes, labeling chemistry, N‐end rule pathway, protein labeling, proteolysis, ubiquitination

## Abstract

- The N‐end rule pathway has emerged as a major system for regulating protein functions by controlling their turnover in medical, animal and plant sciences as well as agriculture. Although novel functions and enzymes of the pathway have been discovered, the ubiquitination mechanism and substrate specificity of N‐end rule pathway E3 ubiquitin ligases have remained elusive. Taking the first discovered *bona fide* plant N‐end rule E3 ligase PROTEOLYSIS1 (PRT1) as a model, we used a novel tool to molecularly characterize polyubiquitination live, in real time.
- We gained mechanistic insights into PRT1 substrate preference and activation by monitoring live ubiquitination using a fluorescent chemical probe coupled to artificial substrate reporters. Ubiquitination was measured by rapid in‐gel fluorescence scanning as well as in real time by fluorescence polarization.
- The enzymatic activity, substrate specificity, mechanisms and reaction optimization of PRT1‐mediated ubiquitination were investigated *ad hoc* instantaneously and with significantly reduced reagent consumption.
- We demonstrated that PRT1 is indeed an E3 ligase, which has been hypothesized for over two decades. These results demonstrate that PRT1 has the potential to be involved in polyubiquitination of various substrates and therefore pave the way to understanding recently discovered phenotypes of *prt1* mutants.

## Introduction

The on/off status of protein function within the cell proteome and the general abundance and specific distribution of proteins throughout the cell compartments are precisely controlled by protein quality control (PQC) mechanisms. These mechanisms ensure that protein functions and activities are directly regulated to maintain the processes critical to the successful survival of any organism. Biochemical analysis of the underlying mechanisms safeguarding proteostatic control is therefore pivotal. Such analysis ranges from the molecular characterization of enzymes involved in PQC and their catalyzed reactions to enzyme substrate and nonsubstrate protein–protein interactions. The so‐called ubiquitin (Ub) 26S proteasome system (UPS) is a master component of PQC, with the key elements being noncatalytic Ub ligases (E3), Ub‐conjugating enzymes (E2), and Ub‐activating enzymes (E1).

To investigate an element conferring substrate specificity, we chose PROTEOLYSIS1 (PRT1) in *Arabidopsis thaliana* as a model E3 ligase, which is a *bona fide* single‐subunit E3 with an unknown substrate portfolio (Bachmair *et al*., 1993; Potuschak *et al*., 1998; Stary *et al*., 2003). Its biological function remains elusive but it presumably represents a highly specific enzyme with E3 ligase function of the N‐end rule pathway of targeted protein degradation, which is part of the UPS. A recent study revealed that, upon cleavage by protease DA1 (‘large’ in chinese 1), the central organ size regulatory protein BIG BROTHER forms a C‐terminal, Tyr‐initiated fragment. Its stability depends on the N‐terminal amino acid Tyr and the function of PRT1 E3 ligase (Dong *et al*., 2017).

**Figure 1:**
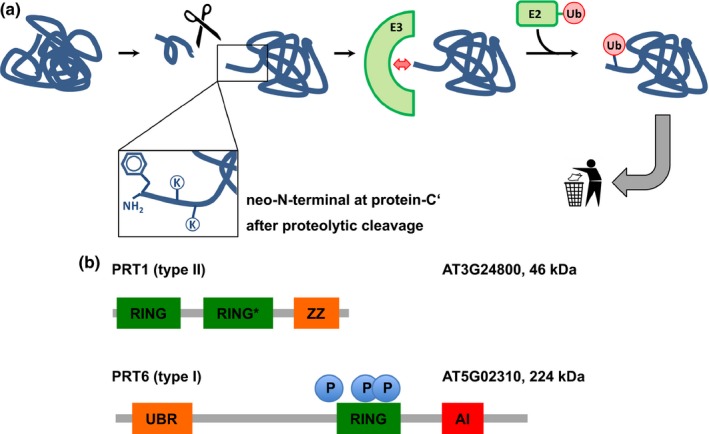
Generation of N‐end rule substrates by proteolytic processing and predicted features of the two *bona fide* plant N‐recognins. (a) Substrates containing N‐degrons can be generated from (pre‐)pro‐proteins as precursor sequences after proteolytic cleavage (indicated by the scissors). The N‐degron shown here comprises a Phe residue as the primary destabilizing residue at the protein‐C′ and internal lysines for polyubiquitination. These N‐degrons can be recognized by N‐end rule E3 Ub ligases (N‐recognins), which, in turn, associate with Ub‐conjugating enzymes (E2) carrying Ub, which was previously activated by E1 enzymes. One possible result of ubiquitination is protein degradation and currently, in the context of the N‐end rule, ubiquitination is assumed to lead to degradation in most cases. (b) The two known *Arabidopsis thaliana* N‐recognins were identified by their function (PROTEOLYSIS1 (PRT1); 46 kDa) and by homology to the so‐called UBR box which is the substrate recognition domain of *Saccharomyces cerevisiae* UBR1p (PRT6; 224 kDa). UBR, structural motif involved in binding type I substrates; RING*, composite domain containing RING (Really Interesting New Gene) and CCCH‐type Zn fingers; ZZ, domain binding two zinc ions, similar to RING; RING, protein–protein interaction domain for E2–E3 interaction; AI, predicted autoinhibitory domain (intramolecular interaction); P, phosphorylation sites confirmed by mass spectrometry (PhosPhAt 4.0; phosphat.uni‐hohenheim.de): pThr1136 and pSer1257 (Roitinger *et al*., 2015) and pThr1335 (Engelsberger & Schulze, 2012).

The N‐end rule relates the half‐life of a protein to its N‐terminal amino acid (Bachmair *et al*., 1986) and causes rapid proteolysis of proteins bearing so‐called N‐degrons, N‐terminal sequences that lead to the degradation of the protein. N‐degrons are created by endoproteolytic cleavage of protein precursors (pro‐proteins) and represent the resulting neo‐N‐termini of the remaining C‐terminal protein moiety, albeit not all freshly formed N‐termini automatically present destabilizing residues (Fig. 1a).

The N‐end rule pathway is a vibrant emerging area of research and has a multitude of functions in all kingdoms (Dougan *et al*., 2010; Varshavsky, 2011; Tasaki *et al*., 2012; Gibbs *et al*., 2014a; Gibbs, 2015). Identified substrates are mainly important regulatory proteins and play key roles in animal and human health (Zenker *et al*., 2005; Piatkov *et al*., 2012; Brower *et al*., 2013; Shemorry *et al*., 2013; Kim *et al*., 2014), plant stress response and agriculture (Gibbs *et al*., 2011, 2014a,b; Licausi *et al*., 2011; Weits *et al*., 2014; de Marchi *et al*., 2016; Mendiondo *et al*., 2016).

In plants, functions of N‐end rule enzymes are associated with central developmental processes including seed ripening and lipid breakdown, hormonal signaling of abscisic acid (ABA), gibberellin and ethylene, seed dormancy and germination (Holman *et al*., 2009; Abbas *et al*., 2015; Gibbs *et al*., 2015), leaf and shoot morphogenesis, flower induction, apical dominance (Graciet *et al*., 2009), and the control of leaf senescence (Yoshida *et al*., 2002). The pathway has been shown to be a sensor for molecular oxygen and reactive oxygen species (ROS) by mediating nitric oxide (NO) signaling and regulating the stress response after hypoxia, for example after flooding and plant submergence (Gibbs *et al*., 2011, 2014b; Licausi *et al*., 2011). A novel plant‐specific class of enzymes, plant cysteine oxidases (PCOs), has been found to be associated with the pathway, indicating the involvement of plant‐specific molecular circuits, enzyme classes and mechanisms in this pathway (Weits *et al*., 2014; White *et al*., 2017). In the moss *Physcomitrella patens*, N‐end rule mutants are defective in gametophytic development (Schuessele *et al*., 2016) and protein targets of N‐end rule‐mediated posttranslational modifications were discovered (Hoernstein *et al*., 2016). Also, in barley (*Hordeum vulgare*), the pathway is connected with development and stress responses (Mendiondo *et al*., 2016). Very recently, a link between N‐end rule function and plant–pathogen response and innate immunity was found (de Marchi *et al*., 2016), shedding light on novel functions of the as yet underexplored process of targeted proteolysis. However, to date, the identity of plant N‐end rule targets still remains obscure and clear evidence from biochemical data obtained in *in vitro* and *in vivo* studies such as N‐terminal subproteomics or enzymatic assays is still lacking.

A novel *in vivo* protein stabilization tool for genetic studies in developmental biology and biotechnological applications, the low‐temperature (‘lt)‐degron’, works in plants and animals by directly switching the levels of functional proteins *in vivo* (Faden *et al*., 2016). The method is based on conditional and specific PRT1‐mediated protein degradation, a process we studied in depth with the generated fluorescently labeled substrate reporter proteins.

N‐degrons are by definition recognized and the corresponding protein ubiquitinated by specialized N‐end rule E3 ligases, so‐called N‐recognins (Sriram *et al*., 2011; Varshavsky, 2011; Tasaki *et al*., 2012; Gibbs, 2015). In plants, only two of these, namely PRT1 and PRT6, are associated with the N‐end rule pathway and are assumed to function as N‐recognins (Fig. 1b). This is in contrast to the high number of proteolytically processed proteins which carry in their mature form N‐terminal amino acids that could potentially enter the enzymatic N‐end rule pathway cascade (Venne *et al*., 2015). Given that there are > 800 putative proteases in the model plant *A. thaliana*, it is likely that the N‐end rule pathway plays an important role in protein half‐lives in a proteome‐wide manner. Examples are found in the METACASPASE9 degradome, that is, the part of the proteome that is associated with degradation (Tsiatsiani *et al*., 2013), or the N‐degradome of *Escherichia coli* (Humbard *et al*., 2013), with a possibly analogous overlap with endosymbiotic plant organelles (Apel *et al*., 2010).

PRT1, compared with the *Saccharomyces cerevisiae* N‐recognin Ubr1 (225 kDa), is a relatively small protein (46 kDa) and is totally unrelated to any known eukaryotic N‐recognin but has functional similarities to prokaryotic homologs (Fig. 1b). It is therefore perceived as a plant pioneer E3 ligase with both diversified mechanisms and function. Artificial substrate reporters based on mouse dihydrofolate reductase (DHFR) comprising an N‐terminal phenylalanine generated via the ubiquitin‐fusion (UFT) technique were used to identify and isolate a *prt1* mutant in a forward mutagenesis screen (Bachmair *et al*., 1993). In the mutant cells and after MG132 treatment, the F‐DHFR reporter construct was shown to be stabilized whereas it was unstable in the untreated wild type (Potuschak *et al*., 1998; Stary *et al*., 2003). *PRT1* was able to heterologously complement a *Saccharomyces cerevisiae ubr1Δ* mutant strain where Phe‐, Tyr‐, and Trp‐initiated β‐galactosidase test proteins were stabilized. These reporters were rapidly degraded in *ubr1Δ* transformed with PRT1 (Stary *et al*., 2003). A new study revealed that cleavage of the E3 ligase BIG BROTHER by protease DA1 forms a C‐terminal, Tyr‐initiated fragment. Its stability depends on the N‐terminal amino acid Tyr and the function of PRT1 E3 ligase (Dong *et al*., 2017). However, to date, there have been no more *in vivo* targets or direct functions associated with PRT1, but, recently, a potential role of PRT1 in plant innate immunity was suggested (de Marchi *et al*., 2016).

The spectrum of N‐termini possibly recognized by plant N‐end rule E3 ligases including PRT1 has not been sufficiently explored. Only Phe‐starting test substrates were found to be stabilized in a *prt1* mutant, whereas initiation by Arg and Leu still caused degradation (Potuschak *et al*., 1998; Stary *et al*., 2003; Garzón *et al*., 2007). In the light of substrate identification, it is crucial to investigate PRT1 mechanisms in more detail, because several posttranslationally processed proteins bearing Phe, Trp and Tyr at the neo‐N‐termini have been found (Tsiatsiani *et al*., 2013; Venne *et al*., 2015) and hence represent putative PRT1 targets. Elucidating the substrate specificity of PRT1 will be an important step forward towards substrate identification and placing PRT1 and the N‐end rule in a biological context.

We established a technique that allows real‐time measurements of ubiquitination using fluorescence scanning of SDS‐PAGE gels and fluorescence polarization. We propose its use as a generic tool for mechanistic and enzymological characterization of E3 ligases as master components of the UPS directing substrate specificity. With a series of artificial test substrates comprising various *bona fide* destabilizing N‐end rule N‐termini, substrate specificity was analyzed and revealed the preference of PRT1 for Phe as a representative of the bulky hydrophobic class of amino acids. The methods commonly used to assay *in vitro* ubiquitination are based on end‐time methods where the reaction is stopped at a given time‐point and analyzed by SDS‐PAGE followed by immunostaining with anti‐Ub vs anti‐target specific antibodies. This detection via western blot often gives rise to the characteristic hallmark of polyubiquitinated proteins, a ‘ubiquitination smear’ or a more distinct ‘laddering’ of the posttranslationally Ub‐modified target proteins. All information about what occurred during the reaction is unknown unless the assay is run at several different time‐points, which drastically increases both experimental time and reagent consumption. Besides the most common methods used for ubiquitination assessment which involve immunodetection with anti‐Ub and anti‐target antibodies, there are few other approaches making use of different reagents. Comparable methods, and their advantages and disadvantages, are listed in Supporting Information Table S1. The novelty offered by the present study is the development of a fluorescence‐based assay that allows real‐time measurement of Ub incorporation in solution, eliminating shortcomings of the existing methods, and thus a more real mechanistic investigation. Our method monitors the ubiquitination process live, in real time, using fluorescently labeled substrate proteins and fluorescence‐based detection assays, namely fluorescence polarization (FP). FP is a spectroscopical technique that allows investigations of the molecular mobility of biomolecules by providing biophysical information on fluorescently labeled molecules. It is used in studies of protein–ligand or protein–protein interactions, polymer formation, proteolysis etc. Its advantage is that it allows the possibility of visualizing molecular binding and dissociation processes in a direct and instantaneous fashion from ‘outside’ without affecting the system. This allows us to acquire real‐time kinetics and information on binding equilibria.

In addition, the protocol was coupled to fast and convenient scanning fluorescence in‐gel detection. This type of assay can be easily adapted for high‐throughput measurements of ubiquitination activity and probably also similar protein modification processes involving changes in substrate molecule properties over time *in vitro*. Rather than merely analyzing enzyme–substrate or protein–protein interactions, the method described here employs FP measurements for the characterization of enzyme activity and parameters affecting the performance of the ubiquitination reaction (Xia *et al*., 2008; Kumar *et al*., 2011; Smith *et al*., 2013).

## Materials and Methods

### Cloning and expression of recombinant proteins

The *Escherichia coli* flavodoxin (Flv; uniprot ID J7QH18) coding sequence was cloned directly from *E. coli* DNA BL21(DE3) and flanked by an N‐terminal triple hemagglutinin (HAT) epitope sequence using the primers Flv_rvs (5′‐TTATTTGAGTAAATTAATCCACGATCC‐3′) and Flv_eK_HAT(oh)_fwd (5′‐CTGGTGCTGCAGATATCACTCTTATCAGCGG‐3′). The X‐eK sequences comprising codons for various N‐terminal amino acids exposed after tobacco etch virus (TEV) cleavage of the expressed X‐eK‐Flv fusion protein were cloned from an eK:HAT template using the primers eK(X)_TEV(oh)_fwd (5′‐GAGAATCTTTATTTTCAGxxx CACGGATCTGGAGCTTG‐3′ with xxx = GTT (for Phe), GGG (for Gly), GAG (for Arg), and GTT (for Leu)) and eK_HAT_flav(oh)_rvs (5′‐CCGCTGATAAGAGTGATATCTGCAGCACCAG‐3′). This sequence contains a TEV protease recognition sequence (ENLYFQ|X, with X being the neo‐N‐terminal after cleavage, i.e. TEV P1' residue) at the N‐terminal of the expressed X‐eK‐Flv fusion protein. In order to attach Gateway attB sites and fuse the PCR products, a PCR was performed using Flv_attB2(oh)_rvs (5′‐GGGACCACTTTGTACAAGAAAGCTGGGTA TCATTATTTGAGTAAATTAATCCACGATCC‐3′) and adapter_tev_fwd (5′‐GGGGACAAGTTTG TACAAAAAAGCAGGCAGGCTTAGAAAACCTGTAT TTTCAGGGAATG‐3′). A Gateway entry clone was generated by BP recombination reaction (Thermo Fisher Scientific, Waltham, MA, USA) with pDONR201 (Thermo Fisher Scientific). All primer sequences are listed in Table S2. A Gateway LR recombination reaction (Thermo Fisher Scientific) into pVP16 (Thao *et al*., 2004) (a kind gift from Russell L. Wrobel, University of Wisconsin, Madison, WI, USA) produced the final construct which consisted of an N‐terminal 8xHis:MBP double affinity tag. The expression vector pVP16::8xHis:MBP:tev:eK:3xHA:Flv was transformed into *E. coli* BL21(DE3) and the fusion protein was expressed by 0.2 mM IPTG (isopropyl β‐d‐1‐thiogalactopyranoside) induction in LB (lysogeny broth) medium for 16 h at 26°C. Cells were harvested via centrifugation (3500 ***g*** at 4°C for 20 min), resuspended in Ni buffer (50 mM sodium phosphate, pH 8.0, and 300 mM NaCl), and treated with 1 mg ml^−1^ lysozyme (Sigma) in the presence of PMSF (sc‐3597; Santa Cruz Biotechnology Inc., Heidelberg, Germany) added to a final concentration of 1 mM followed by sonication (4 min, 40% intensity; 6 min, 60% intensity). The lysate was centrifuged (12 500 ***g*** for 30 min), the supernatant was loaded onto an Ni‐NTA agarose column (Qiagen) equilibrated with Ni buffer, followed by Ni buffer washing, and then the protein was eluted with Ni buffer containing 200 mM imidaziole (Merck, Darmstadt, Germany) and loaded onto amylose resin (New England Biolabs, Ipswich, MA, USA). After washing with amylose buffer (25 mM sodium phosphate, pH 7.8, and 150 mM NaCl), the protein was eluted with amylose buffer containing 10 mM maltose. For the TEV digest, the fusion protein was incubated overnight at 4°C with 0.27 μg μl^−1^ TEV protease, expressed from pRK793 (plasmid 8827; Addgene, Cambridge, MA, USA), in 50 mM phosphate, pH 8.0, 0.5 mM EDTA and 1 mM DTT and loaded onto an Ni agarose column (Qiagen) equilibrated with Ni buffer. The flow‐through containing the tag‐free X‐eK‐Flv substrate was concentrated with an Amicon Ultra‐15 (Merck Millipore, Billerica, MA, USA).

PRT1 was cloned, expressed and purified as described previously (Dong *et al*., 2017).

### Chemical labeling

An incubation of 10 μM purified X‐eK‐Flv was carried out for 1 h at room temperature with a 100 μM concentration of the synthesized thiol reactive fluorogenic labeling dye in 20 mM Tris‐Cl, pH 8.3, 1 mM EDTA and 1 mM tris(2‐carboxy‐ethyl)phosphine (TCEP; Thermo Fisher Scientific). The reaction was stopped with 1 mM cysteine hydrochloride, the unreactive dye was removed using 10‐kDa cut‐off Amicon filters (Merck Millipore) in three successive washing steps, and the labeling efficiency was evaluated on the basis of the fluorescence intensity of the labeled dye using a fluorescence plate‐reader (Infinite M1000; Tecan, Männedorf, Switzerland and the total protein concentration using an infra‐red spectrophotometer (Direct Detect; Merck).

### Chemical synthesis

The detailed synthesis protocols of the labeling probe NBD‐NH‐PEG_2_‐NH‐haloacetamide are described in Methods S1. In brief, the following synthesis steps were accomplished: (1) tert‐butyl {2‐[2‐(2‐aminoethoxy)ethoxy)ethyl}carbamate (NH_2_‐PEG_2_‐NHBoc); (2) NBD‐NH‐PEG_2_‐NHBoc; (3) NBD‐NH‐PEG_2_‐NH_2_ hydrochloride; (4) NBD‐NH‐PEG_2_‐NH‐iodo‐acetamide; (5) NBD‐NH‐PEG_2_‐NH‐iodoacetamide; (6) NBD‐NH‐PEG_2_‐NH‐chloroacetamide.

### Ubiquitination assay and in‐gel fluorescence detection

The X‐eK‐Flv fluorescently labeled substrate (X‐eK‐Flv‐NBD), at a total protein concentration (both labeled and unlabeled) of 3.4 μM, was solved in 25 mM Tris‐Cl, pH 7.4, 50 mM KCl, 5 mM MgCl_2_ and 0.7 mM DTT containing 16 μM Ub from bovine erythrocytes (U6253; Sigma‐Aldrich). For ubiquitination, 2 mM ATP (New England Biolabs), 40 nM E1^15^, 0.31 μM E2 (UBC8)^15^, and 5 nM E3 (8xHis:MBP‐tagged or untagged PRT1) were added to the previously mentioned solution in a final volume of 30 μl and incubated at 30°C for 1 h. The reaction was stopped by adding 5X reductive SDS‐PAGE loading buffer and incubating for 10 min at 96°C followed by SDS‐PAGE. The gels were scanned using fluorescence detection on a Typhoon FLA 9500 biomolecular imager (GE Healthcare, Little Chalfont, Buckinghamshire, UK) with a blue excitation laser (473 nm) LD (laser diode) and an LPB (long‐pass blue) emission filter (510LP), then blotted onto a cellulose membrane and detected with either mouse monoclonal anti‐Ub antibody (Ub (P4D1), sc‐8017; Santa Cruz Biotechnology; 1 : 5000 dilution in blocking solution (150 mM NaCl, 10 mM Tris‐Cl, pH 8, 3% skim milk powder and 0.1% Tween 20)) or mouse monoclonal anti‐HA epitope tag antibody (HA.11, clone 16B12: MMS‐101R; Covance, Princeton, NJ, USA; 1 : 1000 to 1 : 5000 in blocking solution) and goat anti‐mouse IgG‐HRP (1858415; Thermo Scientific™ Pierce™, Waltham, MA, USA; 1 : 2500 to 1 : 5000 dilution in blocking solution). The acquired images of the gels (prior blotting) were analyzed using the Gelanalyzer, online available, free densitometric software (http://Gel.Analyser.com). Thus, one may use the same gel for both in‐gel fluorescence detection followed by blotting and immunodetection.

The same gels that underwent detection via fluorescence scanning were blotted and underwent detection with ECL (enhanced chemiluminescence) without further processing such as stripping. Thus, fluorescent detection can be combined with ECL in one simple workflow. For evaluation of pH dependence, 50 mM Tris‐Cl was used as a buffering agent at pH 6.75, 7.0, 7.5, 8.0, 8.5 and 9.0.

### Real‐time ubiquitination assay using fluorescence polarization

For FP, the reaction mixture (24 μl) containing all the components except the ATP was incubated in a 384‐well microplate (cat. no. 3712 or 3764; Corning, Corning, NY, USA) at 30°C in a M1000 infinite plate reader (Tecan) until the temperature was stable (typically after 4–5 min) and the reaction was triggered by adding 6 μl of 10 mM ATP preheated to 30°C. FP was monitored every 2 min at 562 nm while the excitation wavelength was set to 470 nm. The M1000 fluorescence polarization module was calibrated using 10 nM fluorescein in 10 mM NaOH at FP = 20 mP.

## Results

### PRT1 is an E3 ubiquitin ligase and prefers bulky N‐termini

**Figure 2:**
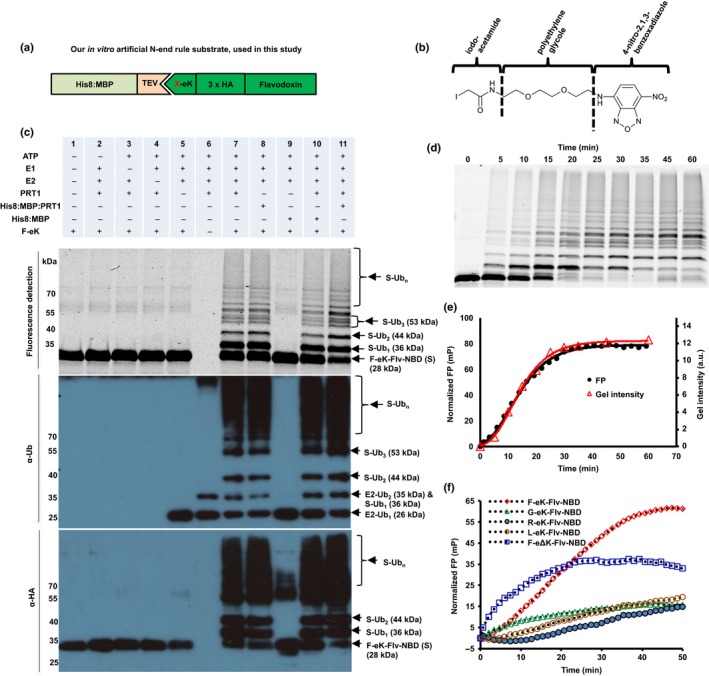
Fluorescent protein conjugates for monitoring *in vitro* substrate ubiquitination in real time. (a) Design of recombinant fusion proteins used as N‐end rule substrates. After tobacco etch virus (TEV) cleavage and removal of the His8:MBP affinity tag, the artificial substrate based on *Escherichia coli* flavodoxin (Flv) is initiated with a neo‐N‐terminal, here Phe (F), Gly (G), Leu (L) or Arg (R). (b) Skeletal formula of the synthesized thiol‐reactive fluorescent compound. The substrate was covalently tagged with the reagent composed of iodoacetamide, polyethylene glycol (PEG) linker and 4‐nitro‐2,1,3‐benzoxadiazole (NBD). The reactive iodine‐containing group on the left couples to the thiol group of internal Cys residues of Flv. NBD serves as a fluorophore with excitation at 470 nm and emission at 520 nm. (c) Detection via fluorescence and immunoblotting of the F‐eK‐Flv‐NBD after *in vitro* ubiquitination. The labeled protein and its ubiquitinated variants were detected via fluorescence scanning directly from the SDS‐PAGE gel followed by western blotting and immunodetection with anti‐HA and anti‐Ub antibodies. Lane 6 shows ubiquitinated E2 and autoubiquitination of PROTEOLYSIS1 (PRT1) as a very high molecular weight ‘smear’. Cleaved PRT1 as well as His8:MBP‐tagged PRT1 were used together with His:UBA1 (E1) and His:UBC8 (E2) (Stegmann *et al*., 2012). (d, e) Kinetic profiles of PRT1‐mediated ubiquitination. F‐eK‐Flv‐NBD ubiquitination was monitored by fluorescence polarization (FP) and in‐gel fluorescence scanning. The S‐shaped kinetic curve is observed in both in‐gel fluorescence scanning detection and fluorescence polarization. (f) N‐terminal specificity evaluated by real‐time ubiquitination detection. Fluorescently labeled R‐eK‐Flv, L‐eK‐Flv, G‐eK‐Flv, F‐eΔK‐Flv and F‐eK‐Flv were comparatively evaluated for their degree of ubiquitination by PRT1.

For the analysis of PRT1 E3 ligase function, that is, recognition of N‐end rule substrates, we used recombinant PRT1 together with generic substrate reagents with novel detection features combining chemically synthesized fluorophores and recombinant ubiquitination acceptors which were used as live protein modification detectors. To describe the N‐terminal amino acid specificity of PRT1, the N‐terminally variable protein parts of the reporters were engineered as N‐terminal His8:MBP fusions comprising a recognition sequence of TEV protease at the junction to the subsequent generic substrate protein moiety (Figs 2a, S1a). Cleavage by TEV gave rise to small C‐terminal fragments of the His8:MBP‐substrate fusions of which the neo‐N‐terminal, that is, the P1' residue of the TEV cleavage site, can be altered to all proteinogenic amino acids except proline (Kapust *et al*., 2002; Phan *et al*., 2002; Naumann *et al*., 2016). For a novel fluorescence‐based approach, we covalently coupled a synthetic fluorescent probe (Fig. 2b) to the artificial substrate protein. The resulting reagent served as the fluorescent protein Ub acceptor in N‐end rule ubiquitination assays. The architecture of the reagent is as follows: after the cleavable His8:MBP tag, eK, part of *E. coli* lacZ (Bachmair *et al*., 1986) followed by a triple hemagglutinin epitope tag (3HA) for immunodetection and an *E. coli* flavodoxin (Flv) were combined. The junctions between His8:MBP and eK encode the N‐termini glycine (Gly (G)), phenylalanine (Phe (F)), arginine (Arg (R)), and leucine (Leu (L)) that become N‐terminally exposed after TEV cleavage. Flv was chosen as a highly soluble and stable protein and includes flavin mononucleotide as a cofactor. Its semiquinone is fluoresent but not stable enough to be used as a fluorophore for detection in its plain form. Therefore, we decided to additionally label the Flv protein. The G/F/L/R‐eK‐Flv constructs contain a single cysteine (Cys101 of Flv) that allowed the labeling of the purified recombinant fusion protein with a novel thiol‐reactive probe that comprises an iodoacetamide‐polyethylene glycol (PEG) linker and the fluorogenic subunit of 4‐nitro‐2,1,3‐benzoxadiazole (NBD; Fig. 2b). We chose the latter in view of its small size compared with other labeling reagents such as large fluorescein moieties and because it can be detected very specifically by both UV absorption and UV fluorescence with low background interference. In principle, if Cys residues are not required for activity, folding or protein interactions, more amenable Cys would lead to higher labeling efficiency. The labeling efficiency was *c*. 30% on average, based on the fluorescence intensity of the labeled substrate, using free NBD as a standard for calibration.

In an *in vitro* ubiquitination assay, we used recombinant UBC8 as a promiscuous E2 conjugating enzyme and UBA1 as an E1 activating enzyme (Stegmann *et al*., 2012) and show here for the first time E3 ligase activity of PRT1 depending on E1, E2 and ATP (Fig. 2c). PRT1 discriminated a substrate by its N‐terminal, aiding the transfer of Ub to the substrate and leading to polyubiquitination. After immunostaining with anti‐Ub antibodies, usually, a typical smear of higher molecular weight compared with the target protein's size is observed or, after probing with target‐specific antibodies, a more or less distinct laddering, also of high molecular weight, becomes evident. These are the common signs for polyubiquitination and a clear laddering was also visualized by fluorescent scanning in our novel approach. We identified distinct subspecies via in‐gel detection (Fig. 2c). A classical end time‐point assay where the reaction was stopped at different reaction time‐points followed by SDS‐PAGE and in‐gel fluorescence detection revealed the kinetics of PRT1 activity using F‐eK‐Flv as a substrate (Fig. 2d).

However, real‐time monitoring of the kinetic profile of the enzymatic reaction is only possible via FP in live detection measurements. The kinetic profile is best fitted with an S‐shaped curve and a growth curve model of logistic type (Richards' equation) rather than exponentially as expected for simple kinetics (Fig. 2e).

It was previously suggested that PRT1 binds to N‐degrons carrying bulky destabilizing residues (Stary *et al*., 2003), but biochemical evidence for that was still lacking. By changing the N‐terminal residue of the X‐eK‐Flv‐NBD substrate, it was possible to reveal that PRT1 indeed discriminates the substrates according to the N‐terminal residue, as expected (Figs 2f, S1b,c). While the substrates carrying G‐, R‐ and L‐initiated N‐termini showed poor ubiquitination, F‐eK‐Flv‐NBD was heavily ubiquitinated. Lysines are the general acceptor sites of Ub transfer from E2 to the substrate and are a requirement for any ubiquitination substrate. Their removal was expected to negatively influence the Ub‐chain formation. There are an additional seven lysine residues present in Flv itself but they are less likely to act as Ub acceptors as they are less accessible (Fig. S1a). While the eK‐based substrate showed the kinetic curve discussed in the previous paragraph, the control F‐eΔK‐Flv substrate with mutated lysines (expected sites of ubiquitination, Lys15 and Lys17, both replaced by Arg) presented a faster initial rate of ubiquitination but FP values of only half the final value (Fig. 2f). This is in good agreement with the in‐gel fluorescence detection, where lower degrees of ubiquitination of F‐eΔK‐Flv, and reduced mono‐ and di‐ubiquitination – but still clear polyubiquitination – were observed (Fig. S1c).

Another observation of the ubiquitination pattern in the in‐gel fluorescence image (using three different independent substrate protein purifications of F‐eK‐Flv‐NBD) was that the tri‐ubiquitinated form presented three distinct subspecies which eventually led to a multitude of other species at a higher level (Fig. S1b). There was only one species of tri‐ubiquitinated F‐eΔK‐Flv‐NBD generated, where two ubiquitination acceptors sites within eK (Lys15 and Lys17) were replaced by Arg (Fig. S1b).

### Fluorescently labeled substrate proteins reveal the mechanism of PRT1‐mediated ubiquitination

**Figure 3:**
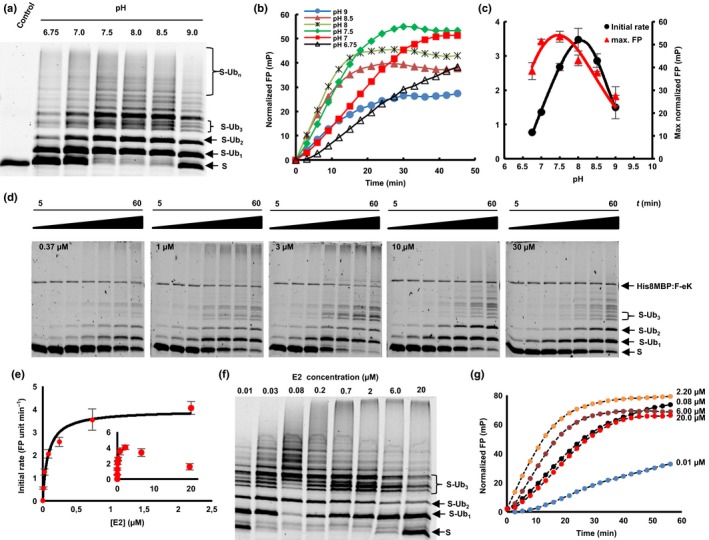
Applications of fluorescent protein conjugates for monitoring pH‐dependent ubiquitination and enzymatic parameters of PROTEOLYSIS1 (PRT1) E3 ligase. (a–c) pH‐dependent ubiquitination of the F‐eK‐Flv substrate. (a) In‐gel detection of F‐eK‐Flv ubiquitinated species after a 1‐h reaction at several pH values demonstrating different patterns of polyubiquitination preferences depending on the pH. (b) Kinetic profiles. (c) Initial rates and maximum end‐time fluorescence polarization (FP) values forming a bell‐shaped distribution depending on the pH. (d–g) PRT1‐mediated ubiquitination of F‐ek‐Flv dependent on the concentration of E2‐conjugating enzyme (UBC8). (d) Time dependence of ubiquitination at several E2 concentrations for the first 60 min at 5 nM PRT1; time scale: 5–60 min. (e) Michaelis–Menten curve plotted using the initial rate from FP data suggests an E2‐driven inhibition effect. (f) Influence of E2 concentration on the ubiquitination pattern evaluated using in‐gel fluorescence scanning and (g) kinetic profiles were obtained using FP measurements, with similar conditions as in (d) but with a 10 times higher concentration of PRT1; that is, 50 nM.

The combination of the proposed two fluorescence‐based methods allowed fast and efficient *in vitro* investigation of the ubiquitination process via the E3 ligase PRT1 and the optimization of the reaction conditions. As a first approach utilizing the real‐time assay in the context of substrate ubiquitination, we studied the effect of changes in pH on the ubiquitination process mediated by PRT1. The pH is an important parameter regulating enzyme activity and is especially important for E3 ligase function as the mechanism implies a nucleophilic attack of the accepting lysine of the substrate on the carbonyl group of the thioester of E2‐Ub. This attack is highly dependent on the pH  < A classical end‐time approach revealed the reaction optimum to be clearly above pH 7 but below pH 9, as indicated by the occurrence of polyubiquitinated species of the fluorescent substrate probe F‐eK‐Flv‐NBD (Fig. 3a). However, using our real‐time FP protocol, we additionally acquired the kinetic profile of the PRT1‐mediated ubiquitination process (Fig. 3b) and the maximum polarization values of this reaction that were reached (Fig. 3c). These correlated with the amount of polyubiquitinated species detected in the SDS‐PAGE gel‐based end‐time experiment (Fig. 3a) and the highest initial rate (Fig. 3c), whereas the latter appears to be different from the reaction optimum according to the detected maximum FP. We also had previously observed that F‐eΔK‐Flv ubiquitination presented a faster initial rate but only half of the final FP (Fig. 2f) and lower degrees of final ubiquitination (Fig. S1c). Both bell‐shaped forms of the pH dependence for the highest initial reaction rate (pH 8.0) and the maximum substrate polyubiquitination rate (pH 7.5) indicated two competing processes that generate a local maximum (Fig. 3c).

A strong decrease of the ubiquitination rate mediated by PRT1 was observed at higher concentrations of the E2‐conjugating enzyme UBC8 (> 2 μM) via both in‐gel fluorescence (Fig. 3d) and FP (Fig. 3e–g). Based on the FP measurements using a concentration of UBC8 of up to 2 μM, the Michaelis‐Menten constant (*K*_M_) of substrate ubiquitination by PRT1 at different E2 concentrations was found to be in the submicromolar range, 0.08 ± 0.01 μM, indicating very tight binding of E2 to PRT1 compared with other RING (Really Interesting New Gene) E3 ligases (Ye & Rape, 2009; Fig. 3e). Moreover, the distribution pattern of the ubiquitinated substrate species at the end of the reaction (Fig. 3f) and the kinetic profiles of ubiquitination (Fig. 3g) are different, depending on the E2 concentration used.

## Discussion

The N‐end rule pathway is a vibrant emerging area of research in plant sciences and agriculture (Gibbs *et al*., 2011, 2014b; Licausi *et al*., 2011; Weits *et al*., 2014; de Marchi *et al*., 2016; Mendiondo *et al*., 2016; reviewed in Gibbs *et al*., 2014a, 2016; Gibbs, 2015; N. Dissmeyer *et al*., unpublished). Taking the first *bona fide* plant N‐end rule E3 Ub ligase PRT1 as a model, we have described a novel tool with which to molecularly characterize polyubiquitination live, in real time, and have used it to gain mechanistic insights into PRT1 substrate preference, activation and functional pairing with an E2‐conjugating enzyme. To date, the activity and function of enzymatic N‐end rule pathway components have only been speculated upon, and the field was lacking investigations at the molecular level. Here, we have provided the first molecular evidence of ubiquitination activity of an E3 ligase candidate for the plant N‐end rule pathway.

In this study, we have demonstrated PRT1 E3 Ub ligase activity and substrate preference by using recombinant PRT1 together with artificial protein substrates in an *in vitro* fluorescence‐based life ubiquitination assay. We found that, first, the reporter construct based on bacterial Flv chemically coupled to NBD (Fig. 2b) works as a ubiquitination acceptor. Second, this reaction reflects substrate specificity and cannot be considered an *in vitro* artifact, as N‐terminal amino acids other than Phe rendered the substrate a weaker target for PRT1 (Figs 2f, S1b,c). Third, our test system allowed description of E3 ligase function and target specificity by using variants of labeled substrates.

Similar experiments are usually based on immunochemical and colorimetric detection, incorporation of radioisotopes such as ^125^I or ^32^P, or fluorescently labeled native or recombinant Ub (Ronchi & Haas, 2012; Melvin *et al*., 2013; Lu *et al*., 2015a,b; Table S1). However, problems of steric hindrance produced by modifying Ub and difficulties in discriminating between auto‐ and substrate ubiquitination if using labeled Ub may occur. Also, artificial experimental set‐ups such as single‐molecule approaches or extreme buffer conditions might not represent or support formation of the required complex ubiquitination machinery (Table S1). Our assay allowed both direct assessment during the actual FP experiment and gel‐based evaluation after completing SDS‐PAGE. This renders protein transfer via western blotting and the subsequent time‐consuming steps of blocking, immunodetection and chemical detection obsolete. The protocol described is rapid and nonradioactive, uses only a small fluorophore as a covalent dye, and works with complete substrate proteins instead of only peptides, and the results produced can be read out live in real time. Moreover, the FP approach provides superimposable kinetic curves with data from classical end time‐point assays, but faster, with higher resolution in time and using fewer reagents. The advantage of a combination of the two fluorescence‐based approaches described, that is, the gel‐based approach and FP, is the possibility of gaining mechanistic insights, which is not possible if only one of the protocols is used. An example is the determination of *K*_M_ and the catalytic rate constant (*k*_cat_) of the interaction of the E3 ligase PRT1 with E2‐conjugating enzymes. This determination included the influence of the E2 concentration on both the ubiquitinated substrate species and the kinetic profile of the ubiquitination reaction.

Using FP coupled to immunoblot analysis, we were able to confirm that PRT1 is an active E3 ligase acting in concert with the E2‐conjugating enzyme UBC8. In a buffer system close to physiological conditions, it could be shown that PRT1 not only monoubiquitinates N‐degron‐containing substrates, but also mediates polyubiquitination without the aid of further cofactors. Therefore, it was ruled out that PRT1 only monoubiquitinates, which was speculated previously (Stary *et al*., 2003). Moreover, the action of a type II N‐recognin as small as PRT1 (46 kDa) is probably sufficient for subsequent target degradation by the proteasome. As PRT1 lacks the conserved ClpS domain that confers affinity to type II substrates in other N‐recognins, the binding mechanism of PRT1 to its substrate remains an intriguing open question.

Using FP‐facilitated real‐time monitoring of the kinetic profile of the PRT1‐mediated ubiquitination, we observed an S‐shaped curve of the reaction (Fig. 2e). One explanation for these kinetics and the presence of an initial lag phase is an increase of the affinity of PRT1 for the monoubiquitinated substrates compared with the nonubiquitinated population. Preferences of E2s and E3s for mono‐ or polyubiquitinated substrates and their influence on ubiquitination velocity were shown previously, but it was also shown that initial ubiquitination greatly enhances the binding affinity of E3s for the substrate in subsequent reactions (Sadowski & Sarcevic, 2010; Lu *et al*., 2015b). Chain elongation (Ub–Ub isopeptide bond formation) can be faster than chain initiation, which might represent the rate‐limiting step of the reaction, rather than an E1–E2‐controlled limiting step. Thus, the chain elongation and chain initiation steps appear to be distinct processes that have distinct molecular requisites, in agreement with previous findings for other E3s (Petroski & Deshaies, 2005; Deshaies & Joazeiro, 2009). The lag phase is reduced if the rate is increased by a higher concentration of PRT1 (Fig. 2e).

The FP‐based assay revealed that the kinetic profile of the ubiquitination was dependent on the position and availability of lysines as Ub acceptor sites, as suggested to be characteristic of N‐degrons (Bachmair & Varshavsky, 1989). By lowering the overall number of available lysines in the F‐eΔK‐Flv‐NBD substrate (two lysines fewer than in X‐eK‐Flv constructs, with 11 Lys in total), the overall ubiquitination was detectably reduced. Differences in the kinetic curves of F‐eK‐Flv and F‐eΔK‐Flv indicated that a reduction in the number of available Lys residues led to a faster initial rate of ubiquitination, whereas the final FP values reached only half the values obtained in the assay applying the substrate with the full set of Lys residues (Figs 2f, S1c). However, the simple gel‐based end‐point assay could not determine if this was attributable to altered velocity of chain initiation vs chain elongation. The initiation per Lys residue was expected to be similar in F‐eK‐ vs F‐eΔK‐Flv substrates, but chain elongation could apparently start more rapidly in F‐eΔK‐Flv. This demonstrated that the presence of E2 together with the particular substrate plays a key role in the formation of the molecular assembly facilitating the ubiquitination process. Already, the intermolecular distance between the E3 ligase and the Ub acceptor lysines of the substrate as well as the amino acid residues proximal to the acceptor lysines determine the progress of the reaction and ubiquitination specificity (Sadowski & Sarcevic, 2010). Taking the slower initiation of polyubiquitination of F‐eK‐Flv into account, the availability of lysines at the N‐terminus might interfere with the monoubiquitination of other, more distal lysines and E3 could remain associated with substrates that are monoubiquitinated at the N‐terminal.

When the F‐eK‐Flv‐NBD substrate fusion protein was subjected to *in vitro* ubiquitination assays, three distinct subspecies of the tri‐ubiquitinated form were detected vs only one form if F‐eΔK‐Flv‐NBD was used (Fig. S1c). This could be explained by the formation of various ubiquitinated isoforms of the substrate by utilizing different lysine side chains as ubiquitination acceptor sites. These could be either within the sequence of eK (e.g. Lys15 and Lys17) or within Flv (e.g. Lys100 and Lys222, which seem structurally more favored according to the structural model; Fig. S1a). This was further supported by the fact that there is only one species of tri‐ubiquitinated F‐eΔK‐Flv‐NBD, where two ubiquitination acceptor sites within eK (Lys15 and Lys17) are replaced by Arg (Fig. S1b).

In the analysis of the influence of pH on the function of PRT1 as an E3 Ub ligase, we documented bell‐shaped forms of pH dependence for the highest initial reaction rate (pH 8.0) and determined the maximum substrate polyubiquitination rate (pH 7.5). These indicated two competing processes that generate a local maximum (Fig. 3c). In the light of recently discussed mechanisms of E3 ligase action (Berndsen & Wolberger, 2014) and the prediction of two RING domains in PRT1 (Stary *et al*., 2003), higher ubiquitination rates with increased pH could be attributable to deprotonation of the attacking lysine side chain of the E2 active site. This would facilitate thioester cleavage between E2 and Ub and thereby mediate Ub transfer to the substrate lysines. A similar effect was observed regarding the influence of the acidic residues in close vicinity to the E2 active site, which also cause deprotonation of the lysine side chain of the incoming substrate (Plechanovova *et al*., 2012). This possibly explains the drastic increase in the initial rate of PRT1 substrate ubiquitination in the range pH 6.8–8 (Fig. 3c). The competing processes leading to the decrease in ubiquitination at pH > 8 could be destabilization of ionic and hydrogen bonds at alkaline pH simply interfering with protein−protein interaction or ATP hydrolysis affecting the Ub charging of E2 by E1. This could also explain the premature leveling of the kinetic curves in the FP measurements at pH > 8 (Fig. 3b) while, at a longer reaction timescale, the maximum FP values would be expected to be the same from pH 6.8 to 7.5.

The apparent *k*_cat_ of the Ub transfer, more precisely the transfer of the first Ub molecule, which is the rate‐limiting step, was found to be 1.30 ± 0.07 s^−1^. This suggested that, on the one hand, PRT1 had a high turnover number as a result of a highly active catalytic center and, on the other hand, that the E2 concentration influences not only the rate of the Ub transfer to the substrate but also the mechanism itself. Possible causes are the two separate and potentially distinctly favored chain initiation and elongation processes mentioned above. These could result in lowering the rate of the initiation step at higher E2 concentrations, as both the kinetic profile and the formation of ubiquitinated species are affected and also the attacking lysines might be structurally differently favored. This is especially suggested by the variable occurrence of the distinct pattern of tri‐ubiquitinated substrate species (Fig. 3d,f), as mentioned above and discussed in other systems (Ye & Rape, 2009).

By using fluorescently labeled substrate proteins in the two approaches described, that is, gel‐based fluorescence scanning after SDS‐PAGE and FP, we were able to investigate the mechanism of PRT1‐mediated ubiquitination and optimize the reaction conditions. The presented work serves as a model for the demonstration of differential mechanisms of substrate recognition and tight interactor binding in the N‐end rule pathway.

PRT1 is a plant pioneer enzyme lacking homologs in the other kingdoms, albeit small and easy to produce in an active form as a recombinant protein, rendering it an exciting candidate for further functional and structural studies of key functions of one branch of the N‐end rule pathway. So far, only three research articles mention work on PRT1: the two first brief descriptions (Potuschak *et al*., 1998; Stary *et al*., 2003) and one recently published study highlighting the role of the N‐end rule pathway – and in particular a novel function for PRT1 – in plant immunity (de Marchi *et al*., 2016). However, to date, the community lacks proofs demonstrating that PRT1 and other E3 candidates are indeed involved in substrate protein ubiquitination.

The tool described here can be adopted by laboratories investigating N‐end rule‐related posttranslational modifications such as deformylation, methionine excision, oxidation, deamidation, arginylation, ubiquitination and degradation. Moreover, we are convinced that it may also be extended to assays for other posttranslational modifications such as phosphorylation and to other E3 Ub ligases as long as at least one native or artificial substrate protein for the modification of interest is known. Because it makes use of chemical labeling of substrate proteins rather than labeling protein modifiers themselves, such as Ub or phosphate, one common reagent can be used for various modification assays. The approach allows one to measure and track posttranslational protein modification live and in a time‐resolved manner and has profound implications for our understanding of the interactions of E3 ligases with substrates and nonsubstrates. Concerning the field of the N‐end rule pathway, this might apply to other candidates for E3 Ub ligases, such as PROTEOLYSIS6 (PRT6) and BIG (AT3G02260), or potential N‐end rule adapter proteins, such as PRT7 (AT4G23860) (Tasaki *et al*., 2005; Garzón, 2008; Talloji, 2011). These experiments will be of great interest in the future because phenotypes of biological importance and genetically determined causalities have been described and need to be substantiated at the molecular level. Therefore, we see potential for a broader impact for ubiquitination research, as it is conceivable that the method is transferable to other E3 ligases and enzyme−substrate pairs. In the course of our studies, we felt that rapid, easy and cheap protocols were lacking for in‐depth biochemical analysis of E3 ligase kinetics, and the same holds true for nonradioactive and sterically noninterfering protocols and those where entire proteins and directly labeled substrates can be applied.

In terms of further applications, the kinetic approach allowed collection of data that can assist in setting up high‐throughput assays, for example for screens of inhibitors and assays assessing the influence of small molecules potentially facilitating or enhancing ubiquitination. In our example, this included testing of the enzymatic parameters of E2–E3 interactions and substrate specificities for PRT1. Similar approaches have used labeling with radionuclides or fluorescent dyes coupled to Ub (Ronchi & Haas, 2012; Melvin *et al*., 2013; Lu *et al*., 2015a,b). The latter covalent modification of Ub with fluorescent moieties is often impractical as these groups can sterically hinder the E1‐catalyzed activation and E2‐dependent transthiolation reactions (Ronchi & Haas, 2012). This in turn can alter the rate‐limiting step. The use of radioactive isotopes requires at least the running of an SDS‐PAGE and gel drying or western blotting followed by autoradiography for hours to days (Table S1). Besides the described *in vitro* methods, several protocols and tools were successfully applied *in vivo*, mainly based on translational fusions of fluorescent proteins to degrons of the Ub fusion degradation (UFD) pathway (Hamer *et al*., 2010; Matilainen *et al*., 2016), the N‐end rule pathway (Speese *et al*., 2003; Faden *et al*., 2016) or both (Dantuma *et al*., 2000). Other methods make use of Ub‐binding systems to achieve various read‐outs (Marblestone *et al*., 2012; Matilainen *et al*., 2013; Table S1).

In conclusion, we describe a system for real‐time measurements of ubiquitination in solution with combined fluorescence scanning of SDS‐PAGE gels and fluorescence polarization. This set‐up was used to establish an artificial substrate protein‐based detection reagent that can be used to obtain important mechanistic insights into the E2−PRT1‐substrate interaction. We demonstrated that PRT1 is indeed involved in polyubiquitination of substrate proteins depending on their N‐terminal amino acids and therefore mechanistically investigated PRT1 as a player of the N‐end rule pathway for the first time.

## Author contributions

A.C.M. performed the ubiquitination reactions and related analysis. E.P. and B.W. designed and synthesized the fluorescent probe, B.W. supervised the chemical synthesis, M.K. established PRT1 ubiquitination reactions, C.N. cloned and purified PRT1, and F.F. cloned the X‐eK‐HAT fragment and performed site‐directed mutagenesis. N.D. and A.C.M. designed the study, wrote the manuscript under consultation with all co‐authors and designed the figures. All authors read and approved the final version of the manuscript.

## Supporting information

Please note: Wiley Blackwell are not responsible for the content or functionality of any Supporting Information supplied by the authors. Any queries (other than missing material) should be directed to the *New Phytologist* Central Office.

**Fig. S1** Modeled structure of the F‐eK‐Flv substrate and PRT1 N‐terminal specificity.
**Table S1** State‐of‐the‐art ubiquitination detection methods
**Table S2** Oligonucleotides used in this study
**Methods S1** Synthesis of the chemical probe NBD‐NH‐PEG_2_‐NH‐haloacetamide.

## References

[nph14497-bib-0001] Abbas M , Berckhan S , Rooney DJ , Gibbs DJ , Vicente Conde J , Sousa Correia C , Bassel GW , Marin‐de la Rosa N , Leon J , Alabadi D *et al* 2015 Oxygen sensing coordinates photomorphogenesis to facilitate seedling survival. Current Biology 25: 1483–1488. 25981794 10.1016/j.cub.2015.03.060 PMC4454774

[nph14497-bib-0002] Apel W , Schulze WX , Bock R . 2010 Identification of protein stability determinants in chloroplasts. Plant Journal 63: 636–650. 20545891 10.1111/j.1365-313X.2010.04268.x PMC2988409

[nph14497-bib-0003] Bachmair A , Becker F , Schell J . 1993 Use of a reporter transgene to generate arabidopsis mutants in ubiquitin‐dependent protein degradation. Proceedings of the National Academy of Sciences, USA 90: 418–421. 10.1073/pnas.90.2.418 PMC45673 11607348

[nph14497-bib-0004] Bachmair A , Finley D , Varshavsky A . 1986 *In vivo* half‐life of a protein is a function of its amino‐terminal residue. Science 234: 179–186. 3018930 10.1126/science.3018930

[nph14497-bib-0005] Bachmair A , Varshavsky A . 1989 The degradation signal in a short‐lived protein. Cell 56: 1019–1032. 2538246 10.1016/0092-8674(89)90635-1

[nph14497-bib-0006] Berndsen CE , Wolberger C . 2014 New insights into ubiquitin E3 ligase mechanism. Nature Structural & Molecular Biology 21: 301–307. 10.1038/nsmb.2780 24699078

[nph14497-bib-0007] Brower CS , Piatkov KI , Varshavsky A . 2013 Neurodegeneration‐associated protein fragments as short‐lived substrates of the N‐end rule pathway. Molecular Cell 50: 161–171. 23499006 10.1016/j.molcel.2013.02.009 PMC3640747

[nph14497-bib-0008] Dantuma N , Lindsten K , Glas R , Jellne M , Masucci M . 2000 Short‐lived green fluorescent proteins for quantifying ubiquitin/proteasome‐dependent proteolysis in living cells. Nature Biotechnology 18: 538–543. 10.1038/75406 10802622

[nph14497-bib-0009] Deshaies RJ , Joazeiro CA . 2009 RING domain E3 ubiquitin ligases. Annual Review of Biochemistry 78: 399–434. 10.1146/annurev.biochem.78.101807.093809 19489725

[nph14497-bib-0010] Dong H , Dumenil J , Lu F , Na L , Vanhaeren H , Naumann C , Klecker M , Prior R , Smith C , McKenzie N *et al* 2017 A novel ubiquitin‐activated peptidase regulates organ size in *Arabidopsis* by cleaving growth regulators that promote cell proliferation and inhibit endoreduplication. Genes & Development, doi: 10.1101/gad.292235.116. 10.1101/gad.292235.116 PMC5322733 28167503

[nph14497-bib-0011] Dougan D , Truscott K , Zeth K . 2010 The bacterial N‐end rule pathway: expect the unexpected. Molecular Microbiology 76: 545–558. 20374493 10.1111/j.1365-2958.2010.07120.x

[nph14497-bib-0012] Engelsberger WR , Schulze WX . 2012 Nitrate and ammonium lead to distinct global dynamic phosphorylation patterns when resupplied to nitrogen‐starved *Arabidopsis* seedlings. Plant Journal 69: 978–995. 22060019 10.1111/j.1365-313X.2011.04848.x PMC3380553

[nph14497-bib-0013] Faden F , Ramezani T , Mielke S , Almudi I , Nairz K , Froehlich MS , Hockendorff J , Brandt W , Hoehenwarter W , Dohmen RJ *et al* 2016 Phenotypes on demand via switchable target protein degradation in multicellular organisms. Nature Communications 7: 12202. 10.1038/ncomms12202 PMC4961840 27447739

[nph14497-bib-0014] Garzón M . 2008 Links between the Ubiquitin‐Proteasome system and cell death pathways in Arabidopsis thaliana. Dissertation, University of Cologne, Cologne, Germany.

[nph14497-bib-0015] Garzón M , Eifler K , Faust A , Scheel H , Hofmann K , Koncz C , Yephremov A , Bachmair A . 2007 *PRT6*/*AT5G02310* encodes an *Arabidopsis* ubiquitin ligase of the N‐end rule pathway with arginine specificity and is not the *CER3* locus. FEBS Letters 581: 3189–3196. 17572409 10.1016/j.febslet.2007.06.005

[nph14497-bib-0016] Gibbs DJ . 2015 Emerging functions for N‐terminal protein acetylation in plants. Trends in Plant Science 20: 599–601. 26319188 10.1016/j.tplants.2015.08.008 PMC4601045

[nph14497-bib-0017] Gibbs DJ , Bacardit J , Bachmair A , Holdsworth MJ . 2014a The eukaryotic N‐end rule pathway: conserved mechanisms and diverse functions. Trends in Cell Biology 24: 603–611. 24874449 10.1016/j.tcb.2014.05.001

[nph14497-bib-0018] Gibbs DJ , Bailey M , Tedds HM , Holdsworth MJ . 2016 From start to finish: amino‐terminal protein modifications as degradation signals in plants. New Phytologist 211: 1188–1194. 27439310 10.1111/nph.14105

[nph14497-bib-0019] Gibbs DJ , Conde JV , Berckhan S , Prasad G , Mendiondo GM , Holdsworth MJ . 2015 Group VII ethylene response factors coordinate oxygen and nitric oxide signal transduction and stress responses in plants. Plant Physiology 169: 23–31. 25944828 10.1104/pp.15.00338 PMC4577381

[nph14497-bib-0020] Gibbs DJ , Lee SC , Isa NM , Gramuglia S , Fukao T , Bassel GW , Correia CS , Corbineau F , Theodoulou FL , Bailey‐Serres J *et al* 2011 Homeostatic response to hypoxia is regulated by the N‐end rule pathway in plants. Nature 479: 415–418. 22020279 10.1038/nature10534 PMC3223408

[nph14497-bib-0021] Gibbs DJ , Md Isa N , Movahedi M , Lozano‐Juste J , Mendiondo GM , Berckhan S , Marin‐de la Rosa N , Vicente Conde J , Sousa Correia C , Pearce SP *et al* 2014b Nitric oxide sensing in plants is mediated by proteolytic control of group VII ERF transcription factors. Molecular Cell 53: 369–379. 24462115 10.1016/j.molcel.2013.12.020 PMC3969242

[nph14497-bib-0022] Graciet E , Walter F , Ó'Maoiléidigh D , Pollmann S , Meyerowitz E , Varshavsky A , Wellmer F . 2009 The N‐end rule pathway controls multiple functions during *Arabidopsis* shoot and leaf development. Proceedings of the National Academy of Sciences, USA 106: 13618–13623. 10.1073/pnas.0906404106 PMC2726413 19620738

[nph14497-bib-0023] Hamer G , Matilainen O , Holmberg CI . 2010 A photoconvertible reporter of the ubiquitin‐proteasome system *in vivo* . Nature Methods 7: 473–478. 20453865 10.1038/nmeth.1460

[nph14497-bib-0024] Hoernstein SN , Mueller SJ , Fiedler K , Schuelke M , Vanselow JT , Schuessele C , Lang D , Nitschke R , Igloi GL , Schlosser A *et al* 2016 Identification of targets and interaction partners of arginyl‐tRNA protein transferase in the moss *Physcomitrella patens* . Molecular & Cellular Proteomics: MCP 15: 1808–1822. 27067052 10.1074/mcp.M115.057190 PMC5083111

[nph14497-bib-0025] Holman T , Jones P , Russell L , Medhurst A , Ubeda Tomas S , Talloji P , Marquez J , Schmuths H , Tung S , Taylor I *et al* 2009 The N‐end rule pathway promotes seed germination and establishment through removal of ABA sensitivity in *Arabidopsis* . Proceedings of the National Academy of Sciences, USA 106: 4549–4554. 10.1073/pnas.0810280106 PMC2649959 19255443

[nph14497-bib-0026] Humbard M , Surkov S , De Donatis G , Jenkins L , Maurizi M . 2013 The N‐degradome of *Escherichia coli*: limited proteolysis *in vivo* generates a large pool of proteins bearing N‐degrons. Journal of Biological Chemistry 288: 28913–28924. 23960079 10.1074/jbc.M113.492108 PMC3789986

[nph14497-bib-0027] Kapust R , Tozser J , Copeland T , Waugh D . 2002 The P1' specificity of tobacco etch virus protease. Biochemical and Biophysical Research Communications 294: 949–955. 12074568 10.1016/S0006-291X(02)00574-0

[nph14497-bib-0029] Kim H , Kim R , Oh J , Cho H , Varshavsky A , Hwang C . 2014 The N‐terminal methionine of cellular proteins as a degradation signal. Cell 156: 158–169. 24361105 10.1016/j.cell.2013.11.031 PMC3988316

[nph14497-bib-0030] Kumar E , Charvet C , Lokesh G , Natarajan A . 2011 High‐throughput fluorescence polarization assay to identify inhibitors of Cbl(TKB)‐protein tyrosine kinase interactions. Analytical Biochemistry 411: 254–260. 21129358 10.1016/j.ab.2010.11.038 PMC3078584

[nph14497-bib-0031] Licausi F , Kosmacz M , Weits DA , Giuntoli B , Giorgi FM , Voesenek LA , Perata P , van Dongen JT . 2011 Oxygen sensing in plants is mediated by an N‐end rule pathway for protein destabilization. Nature 479: 419–422. 22020282 10.1038/nature10536

[nph14497-bib-0032] Lu Y , Lee BH , King RW , Finley D , Kirschner MW . 2015a Substrate degradation by the proteasome: a single‐molecule kinetic analysis. Science 348: 1250834. 25859050 10.1126/science.1250834 PMC4450770

[nph14497-bib-0033] Lu Y , Wang W , Kirschner MW . 2015b Specificity of the anaphase‐promoting complex: a single‐molecule study. Science 348: 1248737. 25859049 10.1126/science.1248737 PMC4449139

[nph14497-bib-0034] Marblestone JG , Larocque JP , Mattern MR , Leach CA . 2012 Analysis of ubiquitin E3 ligase activity using selective polyubiquitin binding proteins. Biochimica et Biophysica Acta 1823: 2094–2097. 22721718 10.1016/j.bbamcr.2012.06.013 PMC3465502

[nph14497-bib-0035] de Marchi R , Sorel M , Mooney B , Fudal I , Goslin K , Kwasniewska K , Ryan PT , Pfalz M , Kroymann J , Pollmann S *et al* 2016 The N‐end rule pathway regulates pathogen responses in plants. Scientific Reports 6: 26020. 27173012 10.1038/srep26020 PMC4865862

[nph14497-bib-0036] Matilainen O , Arpalahti L , Rantanen V , Hautaniemi S , Holmberg CI . 2013 Insulin/IGF‐1 signaling regulates proteasome activity through the deubiquitinating enzyme UBH‐4. Cell Reports 3: 1980–1995. 23770237 10.1016/j.celrep.2013.05.012

[nph14497-bib-0037] Matilainen O , Jha S , Holmberg CI . 2016 Fluorescent tools for *in vivo* studies on the ubiquitin‐proteasome system. Methods in Molecular Biology 1449: 215–222. 27613038 10.1007/978-1-4939-3756-1_12

[nph14497-bib-0038] Melvin A , Woss G , Park J , Dumberger L , Waters M , Allbritton N . 2013 A comparative analysis of the ubiquitination kinetics of multiple degrons to identify an ideal targeting sequence for a proteasome reporter. PLoS ONE 8: e78082. 24205101 10.1371/journal.pone.0078082 PMC3812159

[nph14497-bib-0039] Mendiondo GM , Gibbs DJ , Szurman‐Zubrzycka M , Korn A , Marquez J , Szarejko I , Maluszynski M , King J , Axcell B , Smart K *et al* 2016 Enhanced waterlogging tolerance in barley by manipulation of expression of the N‐end rule pathway E3 ligase PROTEOLYSIS6. Plant Biotechnology Journal 14: 40–50. 25657015 10.1111/pbi.12334 PMC5098238

[nph14497-bib-0040] Naumann C , Mot AC , Dissmeyer N . 2016 Generation of artificial N‐end rule substrate proteins *in vivo* and *in vitro* . Methods in Molecular Biology 1450: 55–83. 27424746 10.1007/978-1-4939-3759-2_6

[nph14497-bib-0041] Petroski MD , Deshaies RJ . 2005 Mechanism of lysine 48‐linked ubiquitin‐chain synthesis by the cullin‐RING ubiquitin‐ligase complex SCF‐Cdc34. Cell 123: 1107–1120. 16360039 10.1016/j.cell.2005.09.033

[nph14497-bib-0042] Phan J , Zdanov A , Evdokimov A , Tropea J , Peters H , Kapust R , Li M , Wlodawer A , Waugh D . 2002 Structural basis for the substrate specificity of tobacco etch virus protease. Journal of Biological Chemistry 277: 50564–50572. 12377789 10.1074/jbc.M207224200

[nph14497-bib-0043] Piatkov K , Brower C , Varshavsky A . 2012 The N‐end rule pathway counteracts cell death by destroying proapoptotic protein fragments. Proceedings of the National Academy of Sciences, USA 109: E1839–E1847. 10.1073/pnas.1207786109 PMC3390858 22670058

[nph14497-bib-0044] Plechanovova A , Jaffray EG , Tatham MH , Naismith JH , Hay RT . 2012 Structure of a RING E3 ligase and ubiquitin‐loaded E2 primed for catalysis. Nature 489: 115–120. 22842904 10.1038/nature11376 PMC3442243

[nph14497-bib-0045] Potuschak T , Stary S , Schlogelhofer P , Becker F , Nejinskaia V , Bachmair A . 1998 PRT1 of *Arabidopsis thaliana* encodes a component of the plant N‐end rule pathway. Proceedings of the National Academy of Sciences, USA 95: 7904–7908. 10.1073/pnas.95.14.7904 PMC20902 9653113

[nph14497-bib-0046] Roitinger E , Hofer M , Kocher T , Pichler P , Novatchkova M , Yang J , Schlogelhofer P , Mechtler K . 2015 Quantitative phosphoproteomics of the *ATAXIA TELANGIECTASIA*‐MUTATED (ATM) and *ATAXIA TELANGIECTASIA*‐MUTATED AND RAD3‐RELATED (ATR) dependent DNA damage response in *Arabidopsis thaliana* . Molecular & Cellular Proteomics: MCP 14: 556–571. 25561503 10.1074/mcp.M114.040352 PMC4349977

[nph14497-bib-0047] Ronchi VP , Haas AL . 2012 Measuring rates of ubiquitin chain formation as a functional readout of ligase activity. Methods in Molecular Biology 832: 197–218. 22350887 10.1007/978-1-61779-474-2_14 PMC3579653

[nph14497-bib-0048] Sadowski M , Sarcevic B . 2010 Mechanisms of mono‐ and poly‐ubiquitination: ubiquitination specificity depends on compatibility between the E2 catalytic core and amino acid residues proximal to the lysine. Cell Division 5: 19. 20704751 10.1186/1747-1028-5-19 PMC2927562

[nph14497-bib-0049] Schuessele C , Hoernstein SN , Mueller SJ , Rodriguez‐Franco M , Lorenz T , Lang D , Igloi GL , Reski R . 2016 Spatio‐temporal patterning of arginyl‐tRNA protein transferase (ATE) contributes to gametophytic development in a moss. New Phytologist 209: 1014–1027. 26428055 10.1111/nph.13656

[nph14497-bib-0050] Shemorry A , Hwang C , Varshavsky A . 2013 Control of protein quality and stoichiometries by N‐terminal acetylation and the N‐end rule pathway. Molecular Cell 50: 540–551. 23603116 10.1016/j.molcel.2013.03.018 PMC3665649

[nph14497-bib-0051] Smith M , Scaglione K , Assimon V , Patury S , Thompson A , Dickey C , Southworth D , Paulson H , Gestwicki J , Zuiderweg E . 2013 The E3 ubiquitin ligase CHIP and the molecular chaperone Hsc70 form a dynamic, tethered complex. Biochemistry 52: 5354–5364. 23865999 10.1021/bi4009209 PMC3856692

[nph14497-bib-0052] Speese S , Trotta N , Rodesch C , Aravamudan B , Broadie K . 2003 The ubiquitin proteasome system acutely regulates presynaptic protein turnover and synaptic efficacy. Current Biology 13: 899–910. 12781128 10.1016/s0960-9822(03)00338-5

[nph14497-bib-0053] Sriram S , Kim B , Kwon Y . 2011 The N‐end rule pathway: emerging functions and molecular principles of substrate recognition. Nature Reviews Molecular Cell Biology 12: 735–747. 22016057 10.1038/nrm3217

[nph14497-bib-0054] Stary S , Yin X , Potuschak T , Schlogelhofer P , Nizhynska V , Bachmair A . 2003 PRT1 of *Arabidopsis* is a ubiquitin protein ligase of the plant N‐end rule pathway with specificity for aromatic amino‐terminal residues. Plant Physiology 133: 1360–1366. 14551326 10.1104/pp.103.029272 PMC281630

[nph14497-bib-0055] Stegmann M , Anderson RG , Ichimura K , Pecenkova T , Reuter P , Zarsky V , McDowell JM , Shirasu K , Trujillo M . 2012 The ubiquitin ligase PUB22 targets a subunit of the exocyst complex required for PAMP‐triggered responses in *Arabidopsis* . Plant Cell 24: 4703–4716. 23170036 10.1105/tpc.112.104463 PMC3531861

[nph14497-bib-0056] Talloji P . 2011 Identification of novel components and links in ubiquitin dependent protein degradation pathways of Arabidopsis thaliana. Dissertation, University of Cologne, Cologne, Germany.

[nph14497-bib-0057] Tasaki T , Mulder L , Iwamatsu A , Lee M , Davydov I , Varshavsky A , Muesing M , Kwon Y . 2005 A family of mammalian E3 ubiquitin ligases that contain the UBR box motif and recognize N‐degrons. Molecular and Cellular Biology 25: 7120–7136. 16055722 10.1128/MCB.25.16.7120-7136.2005 PMC1190250

[nph14497-bib-0058] Tasaki T , Sriram S , Park K , Kwon Y . 2012 The N‐end rule pathway. Annual Review of Biochemistry 81: 261–289. 10.1146/annurev-biochem-051710-093308 PMC3610525 22524314

[nph14497-bib-0059] Thao S , Zhao Q , Kimball T , Steffen E , Blommel P , Riters M , Newman C , Fox B , Wrobel R . 2004 Results from high‐throughput DNA cloning of *Arabidopsis thaliana* target genes using site‐specific recombination. Journal of Structural and Functional Genomics 5: 267–276. 15750721 10.1007/s10969-004-7148-4

[nph14497-bib-0060] Tsiatsiani L , Timmerman E , De Bock PJ , Vercammen D , Stael S , van de Cotte B , Staes A , Goethals M , Beunens T , Van Damme P *et al* 2013 The *Arabidopsis* METACASPASE 9 degradome. Plant Cell 25: 2831–2847. 23964026 10.1105/tpc.113.115287 PMC3784583

[nph14497-bib-0061] Varshavsky A . 2011 The N‐end rule pathway and regulation by proteolysis. Protein Science 20: 1298–1345. 21633985 10.1002/pro.666 PMC3189519

[nph14497-bib-0062] Venne AS , Solari FA , Faden F , Paretti T , Dissmeyer N , Zahedi RP . 2015 An improved workflow for quantitative N‐terminal charge‐based fractional diagonal chromatography (ChaFRADIC) to study proteolytic events in *Arabidopsis thaliana* . Proteomics 15: 2458–2469. 26010716 10.1002/pmic.201500014

[nph14497-bib-0063] Weits D , Giuntoli B , Kosmacz M , Parlanti S , Hubberten H , Riegler H , Hoefgen R , Perata P , van Dongen J , Licausi F . 2014 Plant cysteine oxidases control the oxygen‐dependent branch of the N‐end‐rule pathway. Nature Communications 5: 3425. 10.1038/ncomms4425 PMC3959200 24599061

[nph14497-bib-0500] White MD , Klecker M , Hopkinson R , Weits D , Mueller C , Naumann C , O'Neill R , Wickens J , Yang J , Brooks‐Bartlett J *et al* 2017 Plant cysteine oxidases are dioxygenases that directly Enable arginyl transferase‐catalyzed arginylation of N‐end rule targets. Nature Communications 8: 14690. 10.1038/ncomms14690 PMC5376641 28332493

[nph14497-bib-0064] Xia Z , Webster A , Du F , Piatkov K , Ghislain M , Varshavsky A . 2008 Substrate‐binding sites of UBR1, the ubiquitin ligase of the N‐end rule pathway. Journal of Biological Chemistry 283: 24011–24028. 18566452 10.1074/jbc.M802583200 PMC2527112

[nph14497-bib-0065] Ye Y , Rape M . 2009 Building ubiquitin chains: E2 enzymes at work. Nature Reviews Molecular Cell Biology 10: 755–764. 19851334 10.1038/nrm2780 PMC3107738

[nph14497-bib-0066] Yoshida S , Ito M , Callis J , Nishida I , Watanabe A . 2002 A delayed leaf senescence mutant is defective in arginyl‐tRNA:protein arginyltransferase, a component of the N‐end rule pathway in *Arabidopsis* . Plant Journal 32: 129–137. 12366806 10.1046/j.1365-313x.2002.01407.x

[nph14497-bib-0067] Zenker M , Mayerle J , Lerch M , Tagariello A , Zerres K , Durie P , Beier M , Hulskamp G , Guzman C , Rehder H *et al* 2005 Deficiency of UBR1, a ubiquitin ligase of the N‐end rule pathway, causes pancreatic dysfunction, malformations and mental retardation (Johanson‐Blizzard syndrome). Nature Genetics 37: 1345–1350. 16311597 10.1038/ng1681

