# Supplementary material for: Real‐time detection of N‐end rule‐mediated ubiquitination via fluorescently labeled substrate probes
Source: New Phytol. 2017 Mar 9;217(2):613–24. doi: 10.1111/nph.14497 (PMC5763331; doi:10.1111/nph.14497)
Supplement: Supplementary file 1 — Fig. S1 Modeled structure of the F‐eK‐Flv substrate and PRT1 N‐terminal specificity. Table S1 State‐of‐the‐art ubiquitination detection methods Table S2 Oligonucleotides used in this study Methods S1 Synthesis of the chemical probe NBD‐NH‐PEG2‐NH‐haloacetamide. [file NPH-217-613-s001.pdf]

New Phytologist Supporting Information

Article title: **Real-time detection of PROTEOLYSIS1 (PRT1)-mediated ubiquitination via fluorescently labeled substrate probes**

Authors: Augustin C. Mot, Erik Prell, Maria Klecker, Christin Naumann, Frederik Faden, Bernhard Westermann and Nico Dissmeyer

Article acceptance date: 26 January 2017

The following Supporting Information is available for this article:

Fig. S1 Modeled structure of the F-eK-Flv substrate and PRT1 N-terminal specificity.

Table S1 State-of-the-art ubiquitination detection methods.

Table S2 Oligonucleotides used in this study.

Methods S1 Synthesis of the chemical probe NBD-NH-PEG2-NH-haloacetamide.

Fig. S1 Modeled structure of the F-eK-Flv substrate and PRT1 N-terminal specificity.

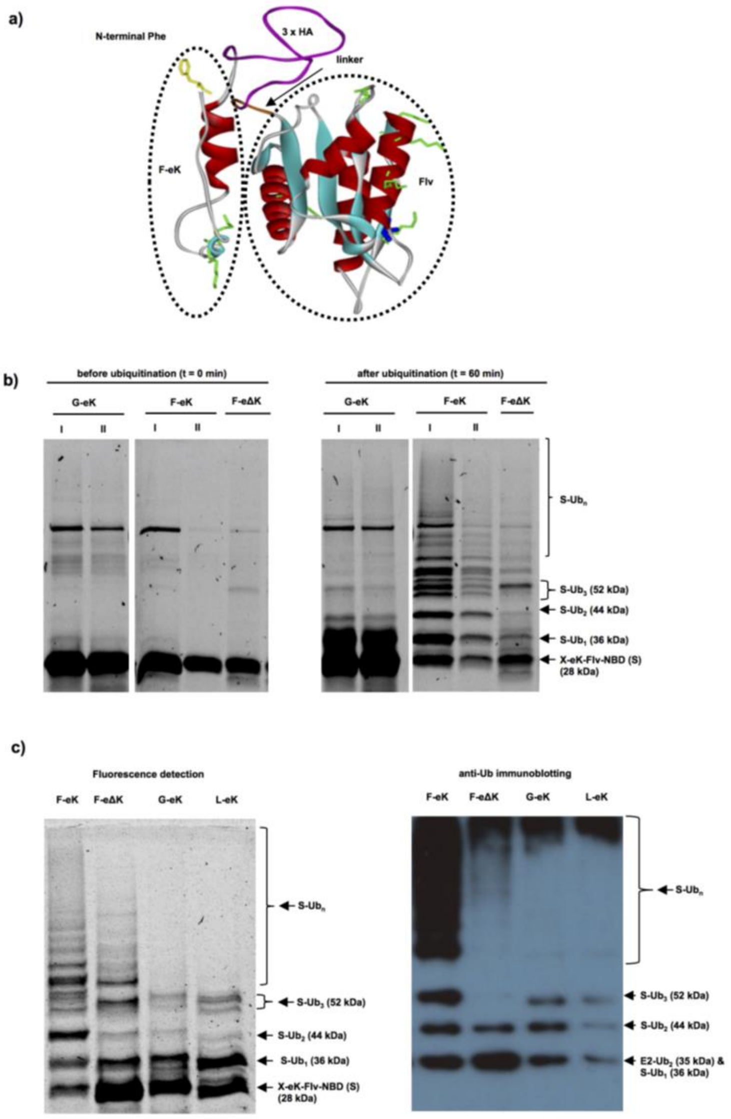

Fig. S1 Modeled structure of the F-eK-Flv substrate and PRT1 N-terminal specificity. (a) Model of F-eK-Flv (using Phyre^2) based on several templates from Protein Data Base (PBD). The modeled structures are superimposable with their original structures or their homologs from the PDB database: Flv (PDB ID: 2M6R) and eK (partly non-structured *E. coli* lacZ repressor fragment, PDB ID: 3EDC). The lysines are depicted in green and the sole cysteine (site of iodoacetamide-NBD coupling) is marked in blue. The 3xHA is colored in violet and the GA linker in orange. The N-terminal Phe residue is marked in yellow. (b, c) PRT1 ubiquitination activity and specificity. (b) Assay conditions and ubiquitination status before and after completed reactions with two independent batches, designated as I and II. (c) *In vitro* ubiquitination assay using the F/G/L-eK-Flv-NBD and F-eΔK-Flv-NBD substrates show different activity depending on the N-termini of the eK-based substrate.

New Phytologist

Table S1 State-of-the-art ubiquitination detection methods.*

Question	Tools and approach	Advantages	Disadvantages	Outcomes	References
general levels of ubiquitination	colorimetric or immunological staining	• established in most laboratories<br>• no specific reagents needed, except anti-Ub antibody or simply Coomassie blue<br>• depending on the protocol, Coomassie staining can be very fast<br>• immunological detection can be strongly amplified using appropriate secondary antibodies and chemical or fluorescent detection methods	• no information about type of ubiquitination<br>• if Coomassie-stained, blotting impossible<br>• if immunodetected, time-consuming	• good estimates of general levels of ubiquitination	many studies use these standard techniques
kinetics of polyubiquitin chain formation	radiolabeled Ub	• rapid<br>• allows detection of ubiquitination kinetics<br>• radiolabeled peptide can be easily quantified<br>• accurate specific radioactivity can be determined and used for precise quantitation of product accumulation<br>• covalent modification of ubiquitin with fluorescent tags or similar moieties is not required since these groups can sterically hinder E1-catalyzed activation of Ub and/or E2-dependent transthiolation which can alter the rate-limiting step	• work with high-energy emitting radionuclides such as 125-I or 32-P<br>• requires running an SDS-PAGE and gel-drying or western blotting followed by autoradiography for hours to days, which would be semi-quantitative only<br>• quantitation might require exposure to phosphoimager screens and scanning<br>• only the radioiodinated protein has been kinetically validated and appeared to be functionally indistinguishable from wild-type non-labeled ubiquitin<br>• not real-time	• functional E1 and E2 concentrations<br>• Michaelis-Menten parameters	Haas *et al*. (1982); Haas & Rose (1982); Huang *et al*. (2005); Ronchi & Haas (2012)
kinetics of polyubiquitin chain formation	fluorescently labeled Ub	• allows analysis of substrate ubiquitination kinetics<br>• non-radioactive, instead use of fluorescein derivatives	• covalent modification of Ub with fluorescent moieties can sterically hinder the E1-catalyzed activation and E2-dependent transthiolation reaction and alter the rate-limiting step<br>• not real-time<br>• peptides are used as substrates rather than entire proteins	• ubiquitination reactions of portable degrons	Melvin *et al*. (2013)
general autoubiquitination activity of an E3	selective polyubiquitin binding proteins, Tandem Ubiquitin Binding Entities (TUBEs), in combination with fluorescently labeled anti-E3 antibody, FRET	• autoubiquitination of E3 ligases in real time<br>• rapid analysis of E3 ligase activity<br>• TUBEs bind selectively to polyubiquitin chains versus mono-ubiquitin thus enabling detection of polyubiquitin chains in the presence of mono-ubiquitin	• tested only for autoubiqutination instead of specific substrates<br>• fluorescently labeled primary antibody required directed against E3 or tagged E3, the latter might affect structure of E3<br>• special detection reagents needed<br>• high background<br>• high levels of fluoride (800 mM) can interfere with mechanism	• dose and time dependent E3 auto-ligase activity	Marblestone *et al*. (2012)
single-molecule approach	fluorescently labeled E3 ligase, Ub and substrate, biotinylated proteins reacted with	• real-time<br>• show kinetics ubiqutination in cell extracts and in purified reaction systems<br>• tracing of individual substrate molecules	• highly artificial: assay runs on a surface containing immobilized peptides/proteins rather than in bulk buffer<br>• too complex for high-throughput analysis	• identification of multiple reaction<br>• intermediates<br>• initial ubiquitination enhances	Lu *et al*. (2015b)

	alexa488— or 647-maleimide, Ub labeled on cysteine	• number of conjugated ubiquitins on a substrate molecule could be measured as the total fluorescence intensity<br>• rates of ubiquitylation and E3-substrate interaction can be measured simultaneously and traced to individual substrate molecules	• background fluctuation from fluorescently labeled but unconjugated free ubiquitin in solution<br>• nonspecific binding of fluorescent species to the slide surface involved use of radioisotopes (here: 33-P)<br>• unclear if capable of identifying multiple reaction intermediates	substrate-binding affinity<br>• characterization of E2-E3 interaction upon ubiquitination<br>• initial ubiquitylation greatly enhances substrate-binding affinity with the E3 in subsequent reactions	
single-molecule kinetic analysis	synthetically constructed ubiquitylated substrates with defined configurations, measured degradation rates of ubiquitylated substrates for electrophoretically resolved species, proteins radio- or fluorescently labeled with DyLight 550 maleimide	• monitor ubiquitination in a single-molecule approach<br>• possibility to distinguish between different configurations of ubiquitination and intermediate steps of degradation	• far for the real mechanism since the natural system involves a dense and interacting populations of bio-molecules not isolated events of single molecules, especially of these type of processes that involves interactions of many enzymes stepwise, concertedly or both.<br>• has to be combined with assays that asses the bulk interactions of the biomolecules<br>• Ub and E3 modified, the kinetic profile is likely to be affected by the structural changes of the Ub<br>• radioisotopes, here 33-P, used to label substrates for in vitro ubiquitylation and degradation assays	• diubiquitin chains provides a more efficient signal. compared to tetra-Ub chains which was suggested until then<br>• ubiquitin chain structures on substrates promote the passage of a bound substrate into the translocation channel on the proteasome	Lu *et al*. (2015a)
global proteasome activity	fluorescent generic substrate chimera (UbG76V-Dendra2), photoconvertible UPS reporter	• live and *in vivo* measurements of proteasome activity	• global proteasome activity is tested, need of pathway specific inhibitors to select routes of ubiquitination	• determination of tissues and cells with high or low proteasome activity, classification of cell types according to general protein turn-over, tool for chemical genetics	Hamer *et al*. (2010); Matilainen *et al*. (2016)
global proteasome activity via the N-end rule	lt-degron fused with GFP	• live and *in vivo* measurements of proteasome activity, can be modulated by temperature-shift and is therefore not constitutively active but tunable	• proteasome activity is tested depending on the N-end rule pathway only, different reporters might be required to assess other routes of ubiquitination	• determination of tissues and cells with high or low proteasome activity, classification of cell types according to general protein turn-over, tool for chemical genetics	Speese *et al*. (2003); Faden *et al*. (2016)
live-imaging of cellular pool of polyubiquitinated proteins	fluorescent generic Ub reporter from polyubiquitin-binding moiety and ZsProSensor	• UIM-domains capture Lys48-linked polyubiquitinated endogenous substrates in the cell, thus stabilizing the reporter<br>• simple read-out: increased fluorescence can be interpreted as an accumulation of polyubiquitinated proteins due to impaired proteasome-mediated degradation	• n.a.; capture tool rather than method for studying kinetics and mechanistics of E3-dependent substrate ubiquitination	• capture cellular pool of polyubiquitin chains from cell lysates<br>• visualizing polyubiquitinated proteins in cells	Matilainen *et al*. (2013); Matilainen, Jha *et al*. (2016)
quantification of Ub/proteasome dependent	short-lived green fluorescent protein variants, use of N-end	• works in vivo in cell culture<br>• simple monitoring by fluorescence intensity<br>• allows correlation between proteasomal	• n.a.; *in vivo* screening tool rather than method for studying kinetics and mechanistics of E3-dependent substrate ubiquitination	• direct targeting of reporter to the Ub/proteasome pathway<br>• indication of G2/M arrest and	Dantuma *et al*. (2000)

proteolysis in living cells	rule and ubiquitin fusion degradation (UFD) signals	degradation and cell growth		apoptosis in cells treated with proteasome inhibitors	
specificity and kinetics of E3 ligase towards predefined set of substrates	fluorescently labeled N-end rule substrates, pathway-specific degradation reporters, X-eK-Flv-NBD	• avoids caveats of labeled Ub such as radioactivity and steric hindrance<br>• simple read-out<br>• compatible for high-throughput analysis<br>• live monitoring via fluorescence polarization (FP)<br>• fast detection of ubiquitination populations via gel-based analysis<br>• comparison of different substrates solely based on N-termini<br>• more physiologically relevant as entire proteins interact in complex<br>• Ub, E1, E2, and E3 remain unmodified	• information entailed by the complex pattern of laddering as seen on western blots is reduced into a single curve<br>• for maximum information, FP has to be combined with in-gel scanning as dual approach<br>• difficult to judge how the pattern (and topology) of ubiquitination relates to the FP signal	• PRT1 is an E3 ligase<br>• specific for hydrophobic bulky N-terminal amino acids<br>• to be used as general assessment of specificity and then for screening and determination of parameters of the reactions live<br>• can potentially be used for analysis of other posttranslational modifications	this study
*excluding anti-Ub or anti-target-based blotting detection assays					

Table S2 Oligonucleotides used in this study.

**Supporting Information Table S1. Oligonucleotides used in this study.**	
**Primer**	**Sequence 5' to 3'**
eK(**F**)_TEV(oh)_fwd	GAGAATCTTTATTTTCA**GTT**CCACGGATCTGGAGCTTG
eK(**G**)_TEV(oh)_fwd	GAGAATCTTTATTTTCA**GGG**TCACGGATCTGGAGCTTG
eK(**R**)_TEV(oh)_fwd	GAGAATCTTTATTTTCA**GAG**ACACGGATCTGGAGCTTG
eK(**L**)_TEV(oh)_fwd	GAGAATCTTTATTTTCA**GTT**GCACGGATCTGGAGCTTG
eK_HAT_Flv(oh)_rvs	CCGCTGATAAGAGTGATATCTGCAGCACCAG
Flv_eK_HAT(oh)_fwd	CTGGTGCTGCAGATATCACTCTTATCAGCGG
Flv_rvs	TTATTTGAGTAAATTAATCCACGATCC
Flv_attB2(oh)_rvs	GGGGACCACTTTGTACAAGAAAGCTGGGTATCATTATTTGAGTAAATTAATCCACGATCC
adapter_tev_fwd	GGGGACAAGTTTGTACAAAAAAGCAGGCAGGCTTAGAAAACCTGTATTTTCAGGGAATG
ss_prt1_tev	GCTTAGAGAATCTTTATTTTCAGGGGATGGCCGAAACTATGAAAGATATTAC
as_prt1_gw	GGGTATCATTCTGTGCTTGATGACTCATTAG
adapter	GGGGACAAGTTTGTACAAAAAAGCAGGCTTAGAGAATCTTTATTTTCAGGGG
prt1_pos2_as	GGGGACCACTTTGTACAAGAAAGCTGGGTATCATTCTGTGCTTGATGA
oh: overhang	

Methods S1 Synthesis of the chemical probe NBD-NH-PEG2-NH-haloacetamide.

Synthesis overview

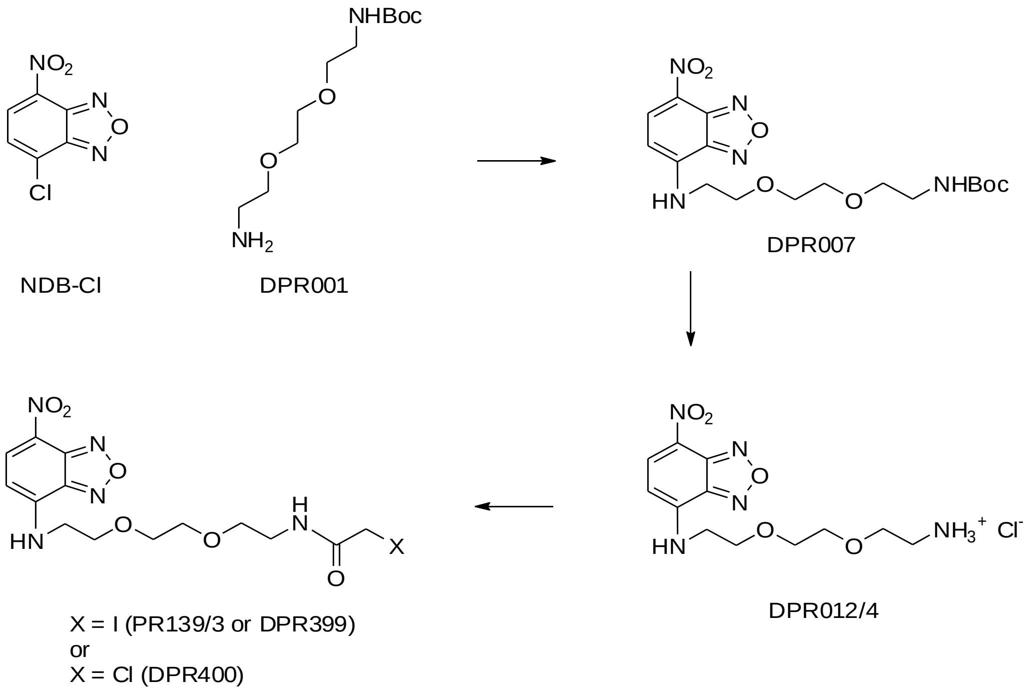

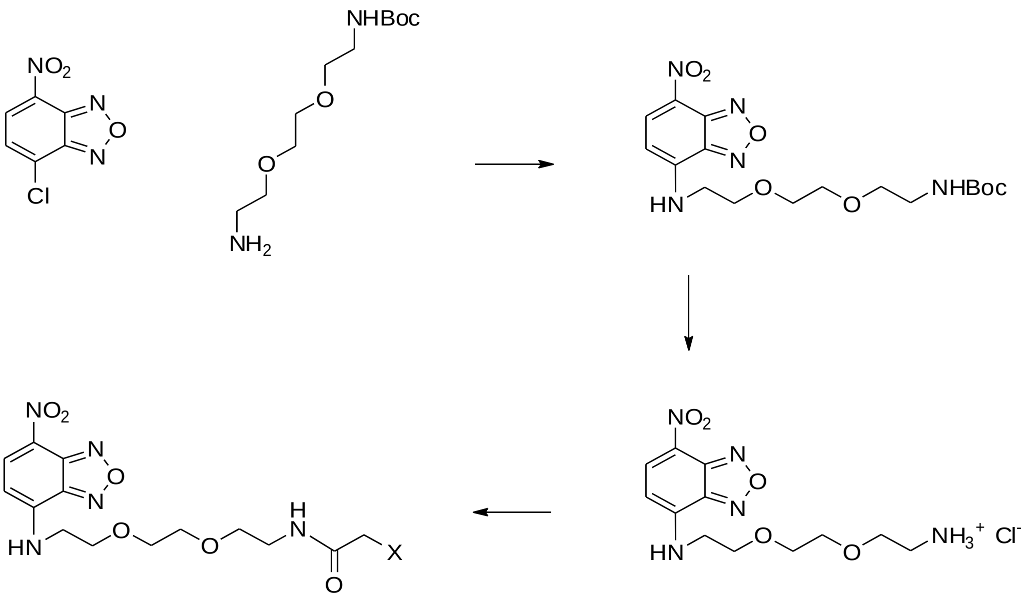
NO2
N
O
N
Cl
NHBoc
O
O
NH2
NO2
HN
O
O
NHBoc
NO2
HN
O
O
NH3+ Cl-
NO2
HN
O
O
H
N
O
X

X = I or X = Cl

tert-butyl {2-[2-(2-aminoethoxy)ethoxy)ethyl}carbamate (NH_2-PEG_2-NHBoc) (DPR001)

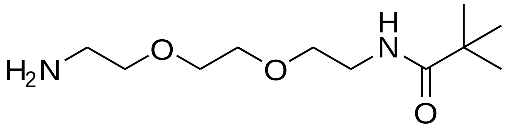

To a solution of 2,2'-(ethylenedioxy)-bis(ethylamine) (50.00 ml, 33.83 mmol; 495.6%) in dry dioxane (190 ml), di-*tert*-butyl dicarbonate (14.90 g, 68.27 mmol, 100%) in dry dioxane (60 mL) was added slowly and the resulting mixture was stirred at 25°C for 12 h. The reaction mixture was filtered, the solvent was removed under reduced pressure and the remaining residue was dissolved in distilled water (300 ml). The aqueous phase was extracted with dichloromethane (3 x 250 ml). Finally, the combined organic phases were dried (Na_2SO_4) and the solvent was removed under reduced pressure to yield *tert*-butyl {2-[2-(2-aminoethoxy)ethoxy)ethyl}carbamate (NH_2-PEG_2-NHBoc) as light yellow oil (16.09 g, 64.8 mmol, 94.9%).

^{1}H NMR (400 MHz; $CDCl_3$) δ: 1.42 (br. s., 2H), 1.42 − 1.46 (m, 9H), 2.87 − 2.90 (m, 2H), 3.32 (m, 2H), 3.52 (m, , 2H), 3.55 (m, , 2H), 3.61 – 3.64 (m, 4H), 5.13 (br. s., 1H) ppm; ^{13}C NMR (100 MHz, $CDCl_3$) δ: 28.4, 40.3, 41.8, 67.1, 70.2, 73.5, 79.2, 156.0 ppm; ESI-MS m/z: 248.7 $[M + H]^+$, 497.4 $[2M + Na^+]^+$; HRMS (ESI) calcd for $C_{11}H_{25}N_2O_4$ 249.1809, found 249.1809.

NBD-NH-PEG$_2$-NHBoc (DPR007/2)

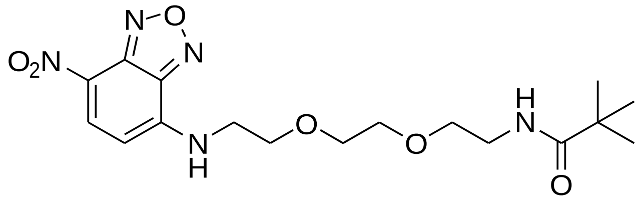

To a suspension of *tert*-butyl {2-[2-(2-aminoethoxy)ethoxy)ethyl}carbamate (1.50 g, 6.04 mmol, 100%) and sodium bicarbonate (1.01 g, 12.08 mmol; 200%) in acetonitrile (30 ml), 4-chloro-7-nitrobenzofurazan (NBD) (1.80 g, 9.06 mmol, 150%) in acetonitrile (30 ml) was added slowly over a period of 2 h and the resulting mixture was stirred at 25°C for 12 h. The reaction mixture was filtered, the solvent was removed under reduced pressure, and the remaining residue was subjected to chromatography (silica gel, methanol / ethyl acetate, 5 : 95) to yield NBD-NH-PEG$_2$-NHBoc as a brown solid (1.89 g, 4.58 mmol, 75.9%).

M.p.: 85 – 86°C; R_F = 0.56 (methanol / ethyl acetate, 5 : 95); ^{1}H NMR (400 MHz; $CDCl_3$) δ [ppm]: 1.42 – 1.45 (m, 9H), 3.31 – 3.37 (m, 2H), 3.54 – 3.56 (m, 2H), 3.58 – 3.60 (m, 2H), 3.61 – 3.71 (m, 4 H), 3.87 (m, 2H), 5.02 (m, 1H), 6.20 (d, *J* = 8.6 Hz, 1H) , 6.88 (m, 1H), 8.49 (d, *J* = 8.6 Hz, 1H); ^{13}C NMR (100 MHz; $CDCl_3$) δ [ppm]: 28.4, 43.6, 68.1, 70.2, 70.2, 70.4, 70.5, 77.2, 98.7, 136.3, 143.9, 144.0, 144.0, 144.3, 155.9; ESI-MS m/z: 410.5 $[M - H]^+$, 434.2 $[M + Na]^+$, 845.4 $[2M + Na]^+$; HRMS (ESI) calcd for $C_{17}H_{25}N_5O_7Na$ 434.1646, found 434.1647.

NBD-NH-PEG$_2$-NH$_2$ hydrochloride (DPR012/4)

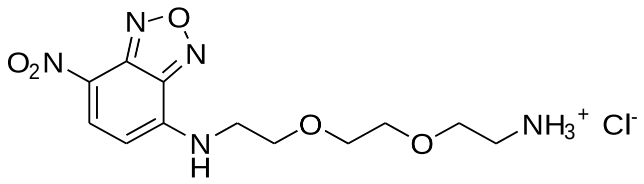

To a solution of NBD-NH-PEG$_2$-NHBoc (2.08 g, 5.06 mmol, 100%) in dry methanol (20 ml), trimethylsilyl chloride (2.70 ml, 21.27 mmol, 500%) was added *via* syringe and the resulting mixture was stirred at 25°C for 12 h. The solvent was removed under reduced pressure. The remaining residue was suspended in diethyl ether (15 ml), filtered and the solid was washed with several portions of diethyl ether, and the remaining solid was dried under reduced pressure to yield NBD-NH-PEG$_2$-NH$_2$ hydrochloride as a brown solid (1.56 g, 5.01 mmol, 98.9%). The crude product was used without further purification.

M.p.: 192 – 193°C; ^{1}H NMR (400 MHz; CD_3OD) δ [ppm]: 3.09 – 3.11 (m, 2H), 3.64 – 3.76 (m, 8H), 3.87 – 3.90 (m, 2 H), 6.19 (d, *J* = 8.4 Hz, 1H), 8.45 (d, *J* = 8.7 Hz, 1H); ^{13}C NMR (100 MHz; CD_3OD) δ [ppm]: 41.5, 41.7, 70.1, 70.3, 70.8, 73.2, 98.8, 123.0, 136.5, 144.1, 144.4, 144.8; ESI-MS m/z: 310.5 $[M - 2H]^+$, 312.3 $[M]^+$; HRMS (ESI) calcd for $C_{12}H_{18}N_5O_5$ 312.1303, found 312.1303.

NBD-NH-PEG$_2$-NH-iodoacetamide (DPR139/3)

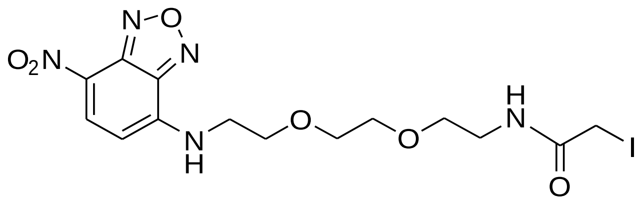

To a solution of NBD-NH-PEG$_2$-NH$_2$ hydrochloride (200 mg, 0.57 mmol; 100%) and DCC (300 mg, 1.45 mmol, 253%) in dry dichloromethane (2.5 ml), iodoacetic acid (250 mg, 1.34 mmol; 234%) was added slowly and the resulting mixture was stirred at 25°C for 12 h. The reaction mixture was filtered, the solvent was removed under reduced pressure, and the remaining residue was subjected to chromatography (silica gel, methanol / ethyl acetate, 10 : 90) to yield NBD-NH-PEG$_2$-NH-iodoacetamide as a brown solid (177.3 mg, 0.37 mmol, 64.9%).

The procedure described above led often to a product, that still contains impurities of *N*,*N*'-dicyclohexylurea. See alternative procedure DPR399.

NBD-NH-PEG$_2$-NH-iodoacetamide (DPR399)

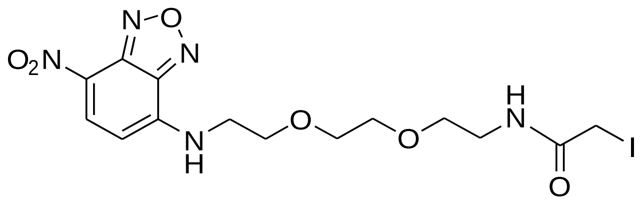

To a solution of NBD-NH-PEG$_2$-NH$_2$ hydrochloride (202.3 mg, 0.65 mmol; 100%) and *N*,*N*'-diisopropylethylamine (134.3 µl, 0.77 mmol, 120%) in dry acetonitril (4.0 ml), iodoacetic anhydride (401.0 mg, 1.13 mmol; 174%) was added slowly and the resulting mixture was stirred at 25°C for 12 h. The solvent was removed under reduced pressure and the remaining residue was subjected to chromatography (silica gel, methanol / ethyl acetate, 10 : 90) to yield NBD-NH-PEG$_2$-NH-iodoacetamide as a brown solid (151.1 mg, 0.32 mmol, 48.5%).

R_F = 0.45 (methanol / ethyl acetate, 10 : 90); ^{1}H NMR (400 MHz; CDCl$_3$) δ [ppm]: 3.50 – 3.54 (m, 2H), 3.62 – 3.65 (m, 2H), 3.69 – 3.71 (m, 8H), 3.73 – 3.76 (m, 2H), 6.21 (d, *J* = 8.7 Hz, 1H), 6.55 (br. s., 1H), 6.95 (br. s., 1H), 8.48 (d, J = 8.6 Hz, 1H); ^{13}C NMR (100 MHz; CDCl$_3$) δ [ppm]: 0.56, 40.1, 43.6, 68.1, 69.4, 70.3, 70.5, 136.4, 143.9, 144.3, 167.1; ESI-MS m/z: 478.3 $[M – H]^+$, 502.1 $[M + Na]^+$ + 981.3 $[2M + Na]^+$; HRMS (ESI (negative modus)) calcd for $C_{14}H_{17}N_5O_6I$ 478.0229, found 478.0222.

NBD-NH-PEG$_2$-NH-chloroacetamide (DPR400)

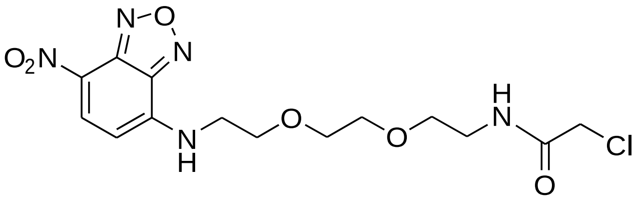

To a solution of NBD-NH-PEG$_2$-NH$_2$ hydrochloride (202.5 mg, 0.65 mmol; 100%) and *N*,*N*'-diisopropylethylamine (134.3 µl, 0.77 mmol, 120%) in dry acetonitril (4.0 ml), chloroacetic anhydride (221.7 mg, 1.30 mmol; 200%) was added slowly and the resulting mixture was stirred at 25°C for 12 h. The solvent was removed under reduced pressure and the remaining residue was subjected to chromatography (silica gel, methanol / ethyl acetate, 10 : 90) to yield NBD-NH-PEG$_2$-NH-chloroacetamid as a brown solid (150.5 mg, 0.39 mmol, 59.7%).

R_F = 0.46 (methanol / ethyl acetate, 10 : 90); ^{1}H NMR (400 MHz; CDCl$_3$) δ [ppm]: 3.54 – 3.58 (m, 2H), 3.64 – 3.75 (m, 8H), 3.87 – 3.90 (m, 2H), 4.06 (m, 2H), 6.20 (d, *J* = 8.7 Hz, 1H), 6.90 (br. s., 1H), 6.98 (br. s., 1H), 8.48 (d, *J* = 8.6 Hz, 1H); ^{13}C NMR (100 MHz; CDCl$_3$) δ [ppm]: 30.51, 42.7, 43.6, 68.1, 69.5, 70.3, 70.5, 136.3, 143.9, 144.3, 166.0; ESI-MS m/z: 386.1 [M – H]$^+$, 410.1 [M + Na]$^+$; HRMS (ESI (negative modus)) calcd for $C_{14}H_{17}N_5O_6Cl$ 386.0873, found 386.0863.

References

Dantuma N, Lindsten K, Glas R, Jellne M, Masucci M. 2000. Short-lived green fluorescent proteins for quantifying ubiquitin/proteasome-dependent proteolysis in living cells. *Nat Biotechnol* **18**: 538-543.

Faden F, Ramezani T, Mielke S, Almudi I, Nairz K, Froehlich MS, Hockendorff J, Brandt W, Hoehenwarter W, Dohmen RJ *et al*. 2016. Phenotypes on demand via switchable target protein degradation in multicellular organisms. *Nat Commun* **7**: 12202.

Haas AL, Rose IA. 1982. The mechanism of ubiquitin activating enzyme. A kinetic and equilibrium analysis. *J Biol Chem* **257**: 10329-10337.

Haas AL, Warms JV, Hershko A, Rose IA. 1982. Ubiquitin-activating enzyme. Mechanism and role in protein-ubiquitin conjugation. *J Biol Chem* **257**: 2543-2548.

Hamer G, Matilainen O, Holmberg CI. 2010. A photoconvertible reporter of the ubiquitin-proteasome system *in vivo*. *Nat Methods* **7**: 473-478.

Huang DT, Paydar A, Zhuang M, Waddell MB, Holton JM, Schulman BA. 2005. Structural basis for recruitment of Ubc12 by an E2 binding domain in NEDD8's E1. *Mol Cell* **17**: 341-350.

Lu Y, Lee BH, King RW, Finley D, Kirschner MW. 2015a. Substrate degradation by the proteasome: a single-molecule kinetic analysis. *Science* **348**: 1250834.

Lu Y, Wang W, Kirschner MW. 2015b. Specificity of the anaphase-promoting complex: a single-molecule study. *Science* **348**: 1248737.

Marblestone JG, Larocque JP, Mattern MR, Leach CA. 2012. Analysis of ubiquitin E3 ligase activity using selective polyubiquitin binding proteins. *Biochim Biophys Acta* **1823**: 2094-2097.

Matilainen O, Arpalahti L, Rantanen V, Hautaniemi S, Holmberg CI. 2013. Insulin/IGF-1 signaling regulates proteasome activity through the deubiquitinating enzyme UBH-4. *Cell Rep* **3**: 1980-1995.

Matilainen O, Jha S, Holmberg CI. 2016. Fluorescent tools for *in vivo* studies on the ubiquitin-proteasome system. *Methods Mol Biol* **1449**: 215-222.

Melvin A, Woss G, Park J, Dumberger L, Waters M, Allbritton N. 2013. A comparative analysis of the ubiquitination kinetics of multiple degrons to identify an ideal targeting sequence for a proteasome reporter. *PLoS ONE* **8**: e78082.

Ronchi VP, Haas AL. 2012. Measuring rates of ubiquitin chain formation as a functional readout of ligase activity. *Methods Mol Biol* **832**: 197-218.

Speese S, Trotta N, Rodesch C, Aravamudan B, Broadie K. 2003. The ubiquitin proteasome system acutely regulates presynaptic protein turnover and synaptic efficacy. *Curr Biol* **13**: 899-910.
